# In silico DFT, ADMET and molecular docking studies of novel coumarin-linked pyrazole and quinoline derivatives as antimicrobial and antioxidant agents

**DOI:** 10.1038/s41598-026-52260-w

**Published:** 2026-05-27

**Authors:** Nargis H. Shaikh, Jay B. Maheta, Darshna K. Lakhnotra, Prince A. Dave, Ila M. Ram, Dharmesh N. Sherathia, Dharmesh K. Katariya, Hardik B. Bhatt, Yogesh O. Bhola, Swanti A. Jain

**Affiliations:** 1 Department of Chemistry, Shri M.P. Pandya Science College, Lunawada, Mahisagar, Gujarat 389230 India; 2 Department of Microbiology and Clinical Technology, Dr. Subhash University, Junagadh, Gujarat 362001 India; 3 Department of Chemistry, Saurashtra University, Rajkot, Gujarat 360005 India; 4 SET Science College & PG Centre, Bhakta Kavi Narsinh Mehta University, Junagadh, Gujarat 362001 India

**Keywords:** Antimicrobial, Coumarin, DFT, Molecular docking, Pyrazole, Biochemistry, Chemistry, Computational biology and bioinformatics, Drug discovery

## Abstract

Ten novel coumarin-based hybrids comprising coumarin–pyrazole (**7a–e**) and coumarin–quinoline (**11a–e**) derivatives were synthesized via multistep reactions and characterized spectroscopically. Solvent optimization identified DMF: Ethanol (1:1) with H_2_SO_4_ as optimal conditions, achieving 88% yield in 2–3 h. Antimicrobial evaluation against four bacterial strains (*Bacillus subtilis*, *Staphylococcus aureus*, *Escherichia coli*, *Acinetobacter baumannii*) and one fungal strain (*Aspergillus niger*) revealed compound **7a** as the most potent (MIC: 25 μg/mL), superior to standard drugs. Antioxidant screening showed that **7a** exhibited excellent activity (IC_50_: 20.23 ± 0.29 mg/mL), comparable to that of ascorbic acid. Molecular docking against three target proteins (4WMZ, 5FNR, 4LOL) demonstrated strong binding affinity, with **7b** showing the highest docking score (− 9.71186 kcal/mol) against 4WMZ. Notably, compound **7b** showed the highest predicted binding affinity in silico, whereas compound **7a** demonstrated the superior experimental antimicrobial activity; this discrepancy underscores that docking scores offer predictive, not confirmatory, evidence of biological potency. DFT calculations (B3LYP/6–311++ G(d,p)) elucidated electronic properties, including HOMO–LUMO gap (3.8640 eV), Mulliken charge distribution, RDG, ELF, LOL, thermodynamic parameters, and global reactivity descriptors. In silico ADMET profiling revealed favorable physicochemical and drug-like properties for most derivatives; however, uniformly high predicted genotoxicity scores (> 0.99) and moderate hERG cardiotoxicity liability were identified, highlighting the need for rigorous experimental safety validation prior to further development. This integrated approach establishes coumarin–pyrazole and coumarin–quinoline hybrids as promising multifunctional therapeutic agents with antimicrobial and antioxidant potential.

**Supplementary Information:**

The online version contains supplementary material available at 10.1038/s41598-026-52260-w.

## Introduction

The rapid emergence of microbial strains that are resistant to most available antimicrobial agents has escalated into a major global health crisis. Antimicrobial peptides (AMPs) natural defense molecules produced by organisms ranging from bacteria to mammals represent a crucial component of innate immunity, a concept first recognized after Fleming identifies lysozyme in 1922. The increasing prevalence of antimicrobial resistance, coupled with the rise in systemic fungal infections, underscores the urgent need for novel therapeutic agents that act through innovative mechanisms^1,2^. Although ketoconazole was one of the earliest successful oral antifungal drugs, growing resistance and toxicity concerns have underscored the demand for safer and more effective alternatives^3,4^.

Heterocyclic compounds remain indispensable in medicinal chemistry because of their structural versatility and broad range of biological activities^5,6^. Among these, pyrazole, coumarin, and quinoline scaffolds have gained significant attention. Pyrazole derivatives exhibit a wide range of pharmacological effects, including anti-inflammatory, antipyretic, antidepressant, antidiabetic, antifungal, and antibacterial properties, and have additional applications in dyes, polymers, corrosion inhibitors, and agrochemicals. Coumarins, naturally occurring phenolic compounds first isolated in 1820, exhibit notable anti-inflammatory, antioxidant, anticancer, and antifungal activities, making them valuable across pharmaceutical and agricultural fields^7,8^. Quinoline, a bicyclic aromatic system comprising fused benzene and pyridine rings, also exhibits diverse biological activities, including anticancer, antimalarial, antibacterial, and anti-inflammatory effects, and serves as the core of many therapeutic agents, dyes, and agrochemicals^9,10^.

**Fig. 1:**
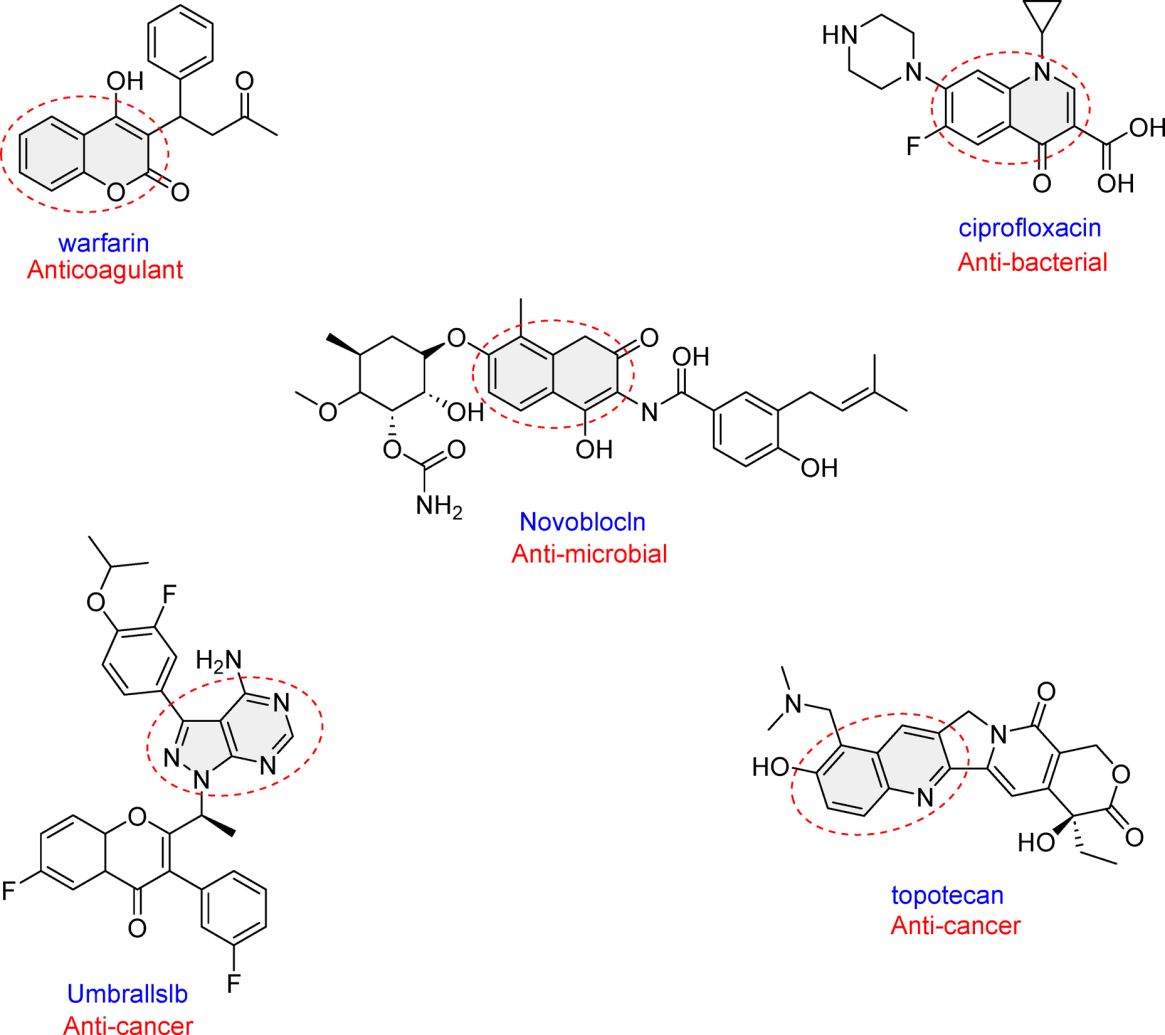
Marketed drugs featuring pyrazole, coumarin and quinoline structural motifs highlight the therapeutic importance of these heterocyclic frameworks.

The therapeutic relevance of these heterocyclic systems is reflected in several well-known drugs, including warfarin and novobiocin (coumarin-based), ciprofloxacin and topotecan (quinoline-based), and umbralisib, which features both pyrazole and coumarin units. These examples emphasize the significance of hybrid heterocyclic scaffolds in contemporary drug development (Fig. 1)^11–13^.

Schiff bases produced through the condensation of aldehydes or ketones with hydrazides are recognized for their ease of synthesis, structural tunability, and broad biological potential. Combining pyrazole, coumarin, and quinoline moieties within a single Schiff base framework provides a strategic approach to enhancing pharmacological activity and exploring new antimicrobial candidates^14^. Indeed, hybridization of the pyrazole scaffold with diverse heterocyclic partners has recently emerged as a productive strategy in antimicrobial drug discovery. Miriyala et al. recently reported benzo[b]thiophene–oxadiazole hybrids bearing pyrazole units with validated antimicrobial, DFT, and ADMET profiles^15^, and other pyrazole-containing molecular hybrids have demonstrated potent antimicrobial and antioxidant activities with favourable pharmacokinetic predictions in recent years^16,17^. The novelty of the present work lies in the concurrent incorporation of the 4-methylcoumarin-7-oxyacetohydrazide linker with either a 3-methyl-5-phenoxypyrazole-4-carbaldehyde or a 2-phenoxyquinoline-3-carbaldehyde unit within a Schiff base framework a structural combination not previously described in the literature designed to exploit the complementary pharmacophoric properties of both heterocyclic systems for enhanced broad-spectrum antimicrobial and antioxidant activity.

In light of these insights, we strategically designed and synthesized a series of coumarin–pyrazole and quinoline hybrid molecules through Schiff base condensation to further investigate their potential as multitarget therapeutic candidates^18^. A comprehensive biological evaluation was carried out to assess their cytotoxic and antimicrobial efficacy. At the same time, density functional theory (DFT) calculations, molecular docking analyses, and ADMET profiling were employed to elucidate their structural features and to gain a deeper understanding of the underlying structure–activity relationships^19,20^.

## Results and discussion

### Chemistry

**Scheme 1:**
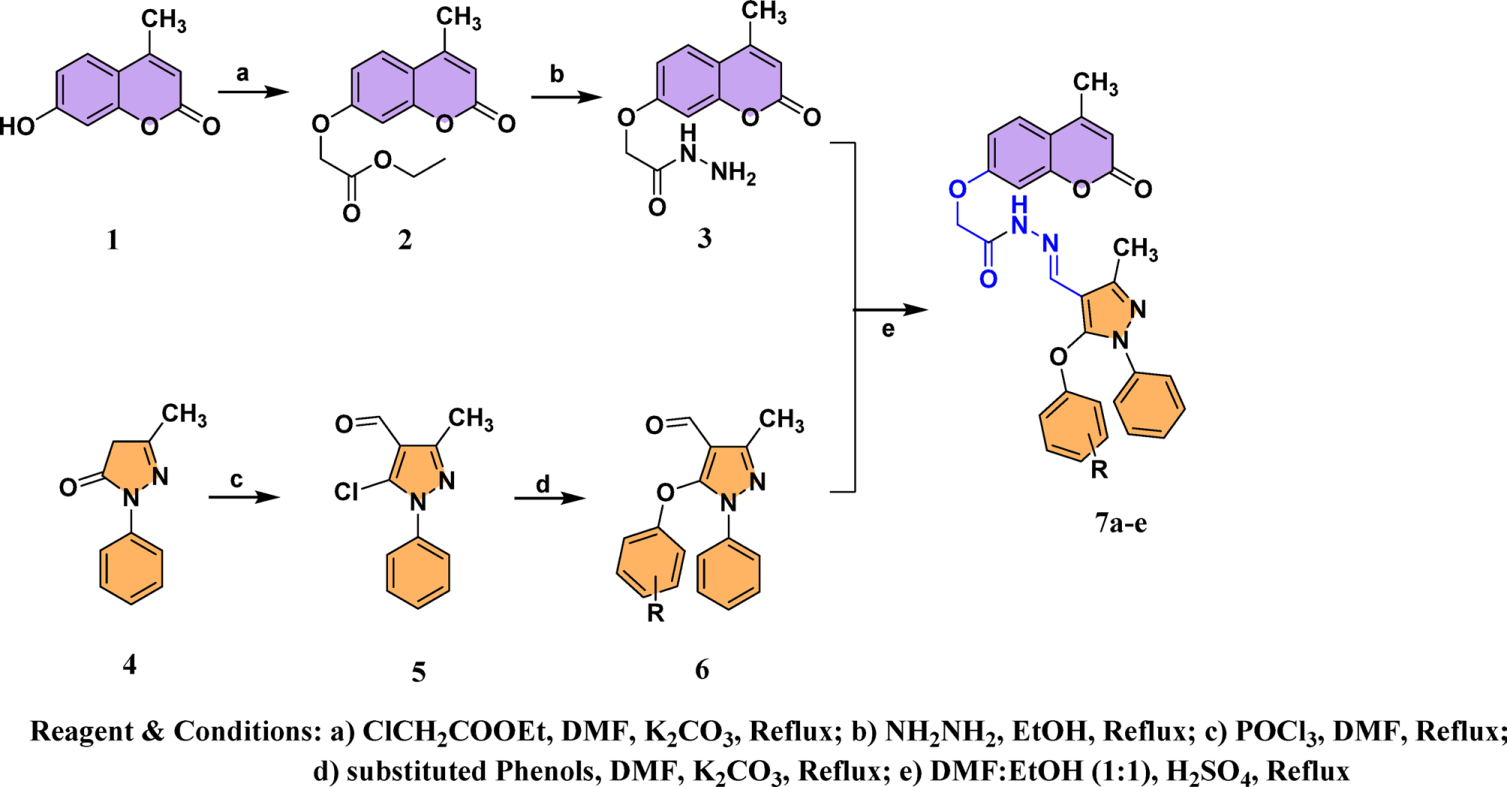
Sequential synthesis of coumarin-pyrazole hybrid (**7a–e**) compounds via parallel synthetic routes.

**Scheme 2:**
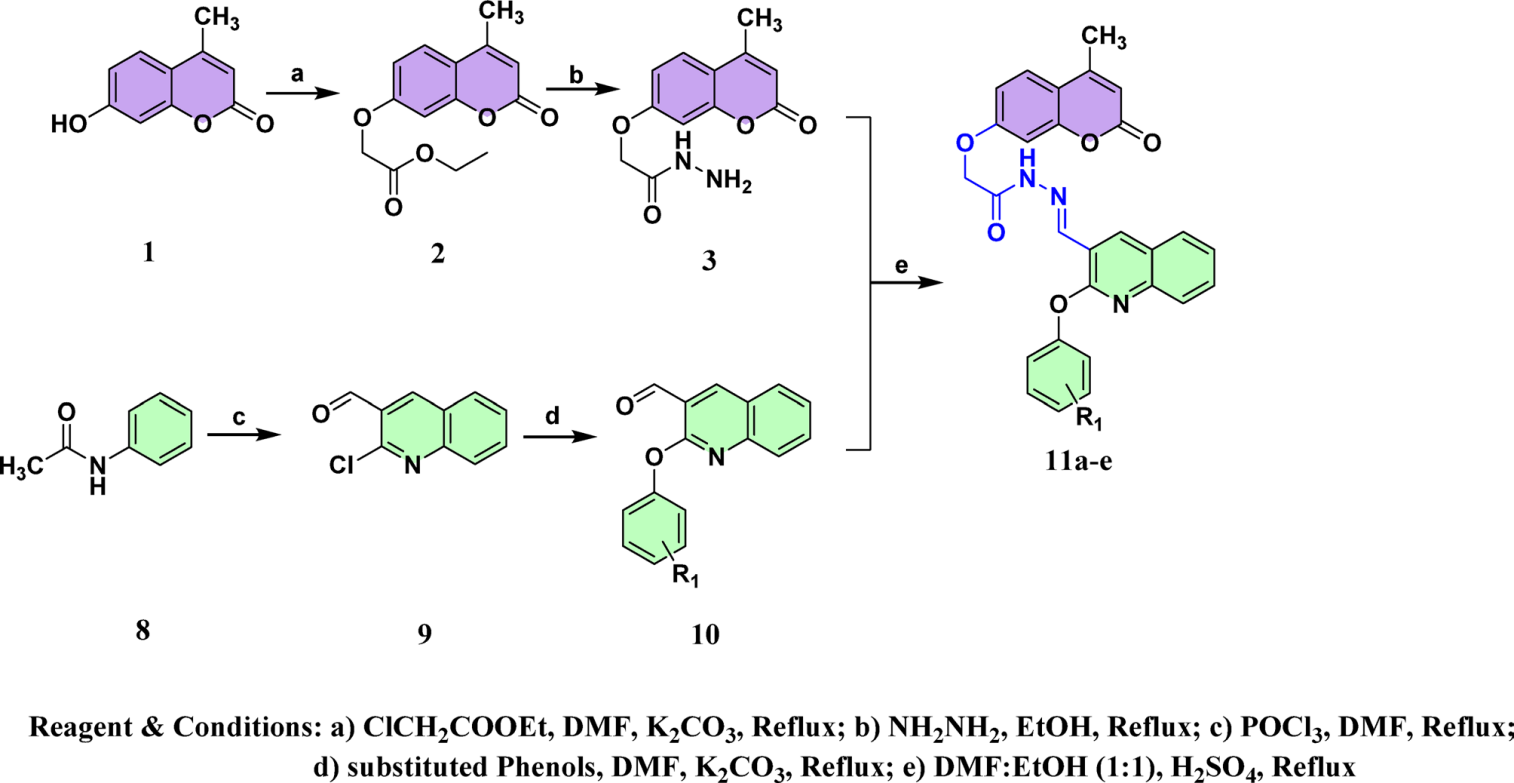
Sequential synthesis of coumarin-quinoline hybrid (**11a–e**) compounds via parallel synthetic routes.

The synthetic pathways for the pyrazole–coumarin and quinoline–coumarin derivatives are outlined in Schemes 1 and 2. Intermediate 2-((4-methyl-2-oxo-2*H*-chromen-7-yl) oxy) acetohydrazide (**3**) was obtained by reacting 4-methyl-7-hydroxycoumarin (**1**) with ethyl chloroacetate in the presence of K_2_CO_3_/DMF to give compound (**2**), followed by treatment with hydrazine hydrate in ethanol. Spectroscopic data confirmed its structure: ^1^H NMR showed coumarin aromatic protons at *δ* 7.01–7.72 ppm, a C-3 singlet at *δ* 6.23 ppm, a C-4 methyl singlet at *δ* 2.41 ppm, and methylene protons at *δ* 4.51 ppm, along with hydrazide signals at *δ* 4.21 ppm (–NH_2_) and *δ* 9.08 ppm (–NH). FT-IR showed N–H stretching (3000–3300 cm^−1^), a lactone C=O band at 1711–1742 cm^−1^, and an amide C=O band near 1670 cm^−1^. ^13^C NMR displayed carbonyl signals at *δ* 160–165 ppm and *δ* 165–175 ppm, aromatic carbons at δ 110–165 ppm, a methylene carbon at *δ* 60–70 ppm, and a methyl carbon at *δ* 18–22 ppm.

Intermediate (**6**) was synthesized via a Vilsmeier–Haack reaction of pyrazolone (**4**) using POCl_3_/DMF to give aldehyde (**5**), followed by reaction with phenol derivatives and 1-naphthol in K_2_CO_3_/DMF. Its ^1^H NMR spectrum showed an aldehyde singlet at *δ* 9.5–10.0 ppm, a methyl singlet at *δ* 2.4–2.6 ppm, and aromatic multiplets at *δ* 6.8–7.6 ppm; FT-IR showed a strong C=O band at 1680–1700 cm^−1^. ^13^C NMR exhibited the aldehyde carbonyl at *δ* 185–191 ppm and aromatic/pyrazole carbons at *δ* 110–165 ppm.

Intermediate (**10**) was synthesized similarly through a Vilsmeier–Haack reaction of acetanilide (**8**) with POCl_3_/DMF to form 2-chloroquinoline-3-carbaldehyde (**9**), followed by reaction with phenol derivatives in K_2_CO_3_/DMF. FT-IR displayed C=O stretching at 1680–1700 cm^−1^ and aldehydic C–H at 2700–2900 cm^−1^. ^1^H NMR showed the aldehyde proton at *δ* 9.5–10.5 ppm and aromatic signals at δ 7.0–8.5 ppm, while ^13^C NMR exhibited the carbonyl carbon at *δ* 185–200 ppm.

Target compounds (**7a–e**) were synthesized by condensing compound (**3**) with compound (**6**) in ethanol/DMF (1:1) using concentrated H_2_SO_4_. A characteristic imine proton appeared at *δ* 8.0–11.5 ppm, with methyl singlets at 2.56 and 2.48 ppm. FT-IR showed C=O stretching at 1712 cm^−1^, C–N at 1192 cm^−1^, and an NH band at 3167 cm^−1^. ^13^C NMR confirmed the carbonyl at 158.1 ppm and –CH=N– at 145.9 ppm. MS showed m/z 508 (M^+^, 2%) and 509.39 (base peak).

Similarly, compounds (**11a–e**) were obtained by condensing compound (**3**) with compound (**10**) under identical conditions. The ^1^H NMR spectrum showed the imine proton at *δ* 8.0–11.6 ppm, with singlets at 2.49 ppm (Me) and 3.78 ppm (OMe). FT-IR displayed bands at 1707 cm^−1^ (C=O), 1298 cm^−1^ (C–N), and 3663 cm^−1^ (NH). ^13^C NMR showed the carbonyl at 158.5 ppm and –CH=N– at 145.6 ppm. MS showed m/z 493 (M^+^, 2%) and 494 (base peak). Additional spectral data are provided in the Supplementary Materials (Tables 1 and 2).

**Table 1:** Synthesis of (*E*)-2-((4-methyl-2-oxo-2*H*-chomen-7-yl) oxy)-*N*-((3-methyl-5-phenoxy-1phenyl-1*H*-pyrazole-4-yl) methylene) acetohydrazide (**7a–e**) and (*E*)-2-((4-methyl-2-oxo-2*H*-chomen-7-yl)oxy)-*N*-((2-phenoxyquinolin-3-yl)methylene)aceto hydrazide (**11a–e**).

Compounds	R	R_1_	Time (h)^a^	Yield (%)^b^
**7a**		–	2.30	75
**7b**		–	2.45	79
**7c**		–	2.50	82
**7d**		–	2.15	85
**7e**		–	2.35	76
**11a**	–		2.20	82
**11b**	–		2.35	78
**11c**	–		2.55	80
**11d**	–		2.45	87
**11e**	–		2.10	79

^a^Reaction completion as visualised by TLC.

^b^Isolated yield.

**Table 2:** Reaction optimization condition for preparation of newly developed Schiff base derivatives (**7a–e**) and (**11a–e**).

Sr No.	Catalyst (mol%)	Solvent and conditions^a^	Time (h)	Temp. (°C)	Yield (%)^b^	Catalyst load (mol%)
1	CH_3_COONa	CH_3_COOH/Reflux	15–17	79	65	(0.0061 mol)
2	–	CH_3_COOH/Reflux	18–22	70	68	–
3	H_2_SO_4_	CH_3_OH/Reflux	8–10	75	75	(0.0094 mol)
4	–	H_2_O	20–25	rt	59	–
**5**	**H**_**2**_ **SO**_**4**_	**DMF:C**_**2**_ **H**_**5**_ **OH (1:1)/Reflux**	**2–3**	70	**88**	(0.0094 mol)

Significant values are in [bold].

^a^Solvent and conditions.

^b^Yield.

### Antimicrobial activity

The synthesized derivatives comprising pyrazole–coumarin (**7a–e**) and quinolone–coumarin (**11a–e**) series were evaluated against two Gram-positive bacteria (*Bacillus subtilis* ATCC 6633, *Staphylococcus aureus* ATCC 6538), two Gram-negative bacteria (*Escherichia coli* 433, *Acinetobacter baumannii* 1425), and one fungal strain (*Aspergillus niger*) using the MIC method. Amoxycillin and Fluconazole served as standards. MIC values ranged from 25 to 200 µg/mL^21^.

Compound **7a** exhibited the most potent broad-spectrum activity with MIC values of 25 µg/mL against *B. subtilis*, *S. aureus*, and *A. niger*, and 50 µg/mL against *E. coli* and *A. baumannii*. It showed comparable activity to Amoxycillin against *S. aureus* (25 µg/mL), superior efficacy against *E. coli* (50 vs. 200 µg/mL), and four-fold better antifungal activity than Fluconazole (25 vs. 100 µg/mL). Compound **7b** demonstrated excellent activity with an MIC of 25 µg/mL against *S. aureus* and 50 µg/mL against other strains. The quinolone–coumarin derivatives (**11a–11e**) showed consistent moderate activity with MIC values of 50–100 µg/mL, particularly effective against *S. aureus* (50 µg/mL) and *A. niger* (50–100 µg/mL). All compounds exhibited superior activity against *E. coli* compared to Amoxycillin (Table 3).

**Table 3:** Antibacterial and antifungal activity (MIC) of synthesized compounds **7a–e** and **11a–e** compared with standard drugs (fluconazole and amoxycillin)^a^.

Compd.	*B. subtilis* (ATCC 6633) µg/mL	*S. aureus* (ATCC 6538) µg/mL	*E. coli* (433) µg/mL	*A. baumannii* (1425) µg/mL	*A. niger* µg/mL
**7a**	25	25	25	50	25
**7b**	50	25	50	50	50
**7c**	200	50	100	50	50
**7d**	50	100	100	100	50
**7e**	50	100	100	100	100
**11a**	200	50	100	50	50
**11b**	100	50	100	50	50
**11c**	100	50	100	100	50
**11d**	100	50	100	100	100
**11e**	100	50	50	100	50
Amoxycillin	12.5	25	200	25	–
Fluconazole	–	–	–	–	100

^a^Data were expressed as mean ± SD of three experiments, – represents no activity.

#### Structure–activity relationship (SAR)

The antimicrobial activity of pyrazole–coumarin (**7a–e**) and quinolone–coumarin (**11a–e**) hybrids was significantly influenced by the nature of aromatic substituents attached to the heterocyclic cores. Both series share a 4-methyl coumarin moiety linked through a carbohydrazide bridge, differing in the pyrazole (**7a–e**) versus quinoline (**11a–e**) ring systems.

#### Pyrazole–coumarin series (7a–e)

Compound **7a** with a phenoxy substitution exhibited the most potent activity (MIC 25–50 µg/mL), attributed to optimal lipophilicity, which facilitates enhanced membrane penetration, and to the oxygen atom, which enables additional hydrogen bonding with biological targets. The naphthyl derivative **7b** demonstrated excellent activity (MIC 25–50 µg/mL), with the extended π-system providing favorable π–π stacking interactions and DNA intercalation capabilities. Tolyl-substituted compounds **7c** and **7d** showed moderate activity (MIC 50–200 µg/mL), with positional isomerism affecting activity patterns **7c** displayed preferential activity against *S. aureus* and *A. baumannii,* while **7d** exhibited more uniform efficacy. Compound **7e** bearing an electron-withdrawing nitro group showed reduced activity, particularly against *B. subtilis* (MIC 200 µg/mL), as the nitro substituent decreases electron density and membrane permeability. However, moderate activity against *S. aureus* and *A. niger* was retained.

#### Quinoline–coumarin series (11a–e)

The quinoline derivatives (**11a–e**) exhibited consistent moderate activity (MIC 50–100 µg/mL). Compounds **11a–c** with phenoxy and tolyl substituents exhibited uniform profiles across bacterial strains and showed excellent antifungal activity (50 µg/mL). The larger, rigid quinoline system provides strong π–π stacking and metal-chelation capabilities but imposes conformational constraints that affect optimal binding. Compounds **11d** and **11e** demonstrated improved activity against *E. coli* (50–100 µg/mL), superior to Amoxycillin (200 µg/mL), suggesting the quinoline scaffold confers selective advantages against Gram-negative bacteria.

#### Comparative analysis

The pyrazole–coumarin series generally outperformed quinoline derivatives, particularly against Gram-positive bacteria. The nitrogen-rich pyrazole core provides superior hydrogen-bonding and metal-chelation capabilities, which are crucial for enzyme inhibition and DNA binding. All derivatives demonstrated remarkable antifungal activity (MIC 25–100 µg/mL), equal to or superior to Fluconazole, indicating the coumarin-carbohydrazide scaffold possesses inherent antifungal properties. The SAR reveals that electron-donating/neutral lipophilic substituents enhance activity, electron-withdrawing groups reduce potency, and the pyrazole core offers superior antimicrobial efficacy over quinoline. Compound **7a** represents the optimal structural motif, combining favorable electronic properties, lipophilicity, and multiple pharmacophoric features for potent broad-spectrum antimicrobial action.

### Antioxidant assay

The DPPH method was utilized to assess the free radical scavenging capacity of the newly synthesized compounds (**7a–e** and **11a–e**)**.** The freshly prepared solution displays a rich blue color, with an absorption peak at 517 nm. The presence of an antioxidant in the solution typically diminishes the intense blue color^22^. All compounds demonstrated varying antioxidant activities when compared to the standard Ascorbic acid. The differences observed in the DPPH scavenging capability can be attributed to the influence of different substituents. Compounds featuring a phenoxy group showed greater radical-scavenging activity. Among the tested compounds, **7a** and **7b** showed vigorous antioxidant activity, with the lowest IC50 values (20.23 ± 0.29 and 24.20 ± 0.64 mg/mL, respectively). In contrast, conjugates **7c**, **7d, 11a**, **11b**, **11c** and **11d** showed moderate scavenging ability, but compounds **7e** and **11e** with a nitro group exhibited lower efficacy against DPPH radicals (Table 4).

**Table 4:** In vitro antioxidant activity of synthesized compounds **7a–e** and **11a–e** evaluated by DPPH radical scavenging assay ^b^.

Compounds	Scavenging activity (IC_50_ mg/mL)
**7a**	20.23 ± 0.29
**7b**	24.20 ± 0.64
**7c**	27.16 ± 0.72
**7d**	32.44 ± 0.29
**7e**	45.40 ± 0.87
**11a**	28.60 ± 0.35
**11b**	37.65 ± 0.21
**11c**	39.92 ± 0.23
**11d**	34.85 ± 0.47
**11e**	47.43 ± 0.50
Ascorbic acid	25.80 ± 0.94

Each value is expressed as mean ± SD of three replicates; Ascorbic acid was used as a standard.

### Molecular docking results

**Fig. 2:**
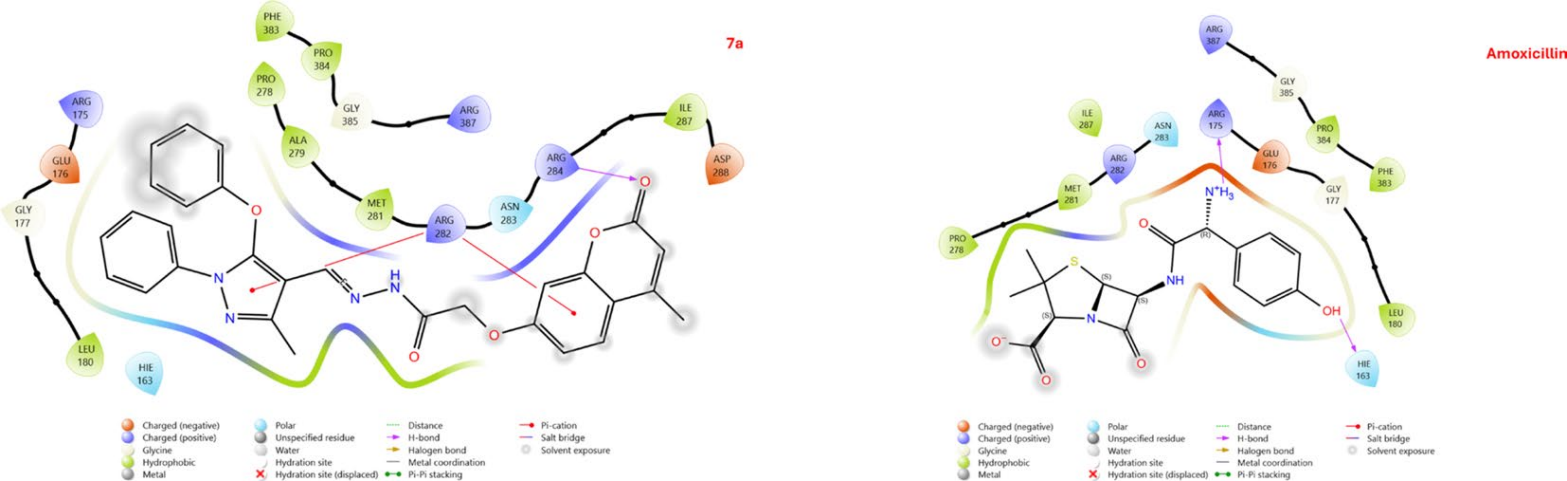
Comparative 2D docking analysis of compound **7a** and amoxicillin with 4LOL protein.

Among all synthetic derivatives, compound **7a** demonstrated a moderate binding affinity, with a binding energy of − 4.0 kcal/mol against the 4LOL receptor, which was higher than that of the standard drug amoxicillin. Amoxicillin formed two hydrogen bonds ARG A:175, HIE A:163 and hydrophobic interactions with PRO A:278, MET A:281, ILE A:287, PRO A:384, PHE A:383, LEU A:180 In contrast, **7a** proven a markedly improved binding energy of –4.6 kcal/mol, forming hydrogen bonds with ARG A:284 along with hydrophobic contacts involving LEU A:180, PRO A:278, ALA A:279, MET A:281, PHE A:383, PRO A:384, amd ILE A:287 (Fig. 2; Table 5).

**Table 5:** Binding interaction of compound **7a** with (PDB ID: 4LOL).

Interacting amino acids					
Compound code	Docking score (kcal/mol)	Hydrophobic interactions	Polar interactions	H-bond	Others
**7a**	− 4.61564	LEU 180, PHE 383, PRO 384. PRO 278, ALA 279, MET 281, ILE 287	ASN 283, HIE 163	ARG 284, (H-bond with coumarin lactone)	ARG 175, ARG 387, (charged positive), ASP 288, GLU 176, (charged negative) GLY 177, GLY 385, ARG 282 (Pi-cation with pyrazole and coumarin)
Amoxicillin	− 4.00365	PRO 278, MET 281, ILE 287, PRO 384, PHE 383, LEU 180	ASN 283	ARG 175, HIE 163 (H-bond with NH_3_ and OH, respectively	ARG 387, ARG 282 (charged positive), GLU 176 (charged negative), GLY 177, GLY 385

**Fig. 3:**
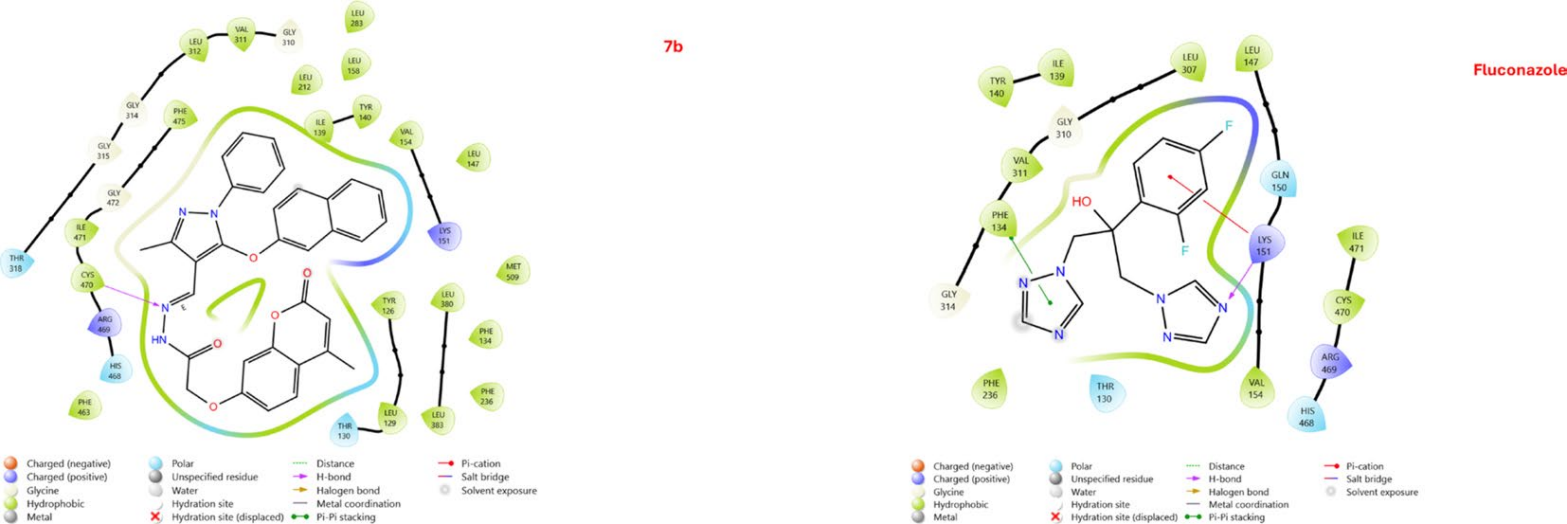
2D molecular docking interactions of compound **7b** and standard drug fluconazole with the 4WMZ protein target.

For the 4WMZ receptor, the standard pharmaceutical agent fluconazole. exhibited a binding energy of − 6.6 kcal/mol. It established hydrogen bonds with LYS A:151 and engaged in hydrophobic interactions with PHE A:236, VAL A:154, CYS A:470, ILE A:471, LEU A:147, LEU A:307, ILE A:139, TYR A:140, VAL A:311, and PHE A:134. In contrast, compound **7b** demonstrated a binding energy of − 9.7 kcal/mol, forming hydrogen bonds with CYS A:470 and hydrophobic interactions with PHE A:463, ILE A:471, PHE A:475, LEU A:312, VAL A:311, LEU A:212, LEU A:158, LEU A:283, TYR A:140, ILE A:139, VAL A:154, LEU A:147, MET A:509, PHE A:134, PHE A:236, LEU A:380, LEU A:383, LEU A:129, and TYR A:126 (Fig. 3; Table 6).

**Table 6:** Binding interaction of compound **7b** with (PDB ID: 4WMZ).

Interacting amino acids					
Compound code	Docking score (kcal/mol)	Hydrophobic interactions	Polar interactions	H-bond	Others
**7b**	− 9.71186	PHE 463, ILE 471, PHE 475, LEU 312, VAL 311, LEU 283, LEU 158, LEU 212, TYR 140, ILE 139, VAL 154, LEU 147, MET 509, PHE 134, PHE 236, LEU 383, LEU 380, TYR 126, LEU 129	THR 318, HIS 468, THR 130	CYS 470 (H-bond with Schiff base group)	ARG 469, LYS 151 (charged positive), GLY 315, GLY 314, GLY 472, GLY 310
Fluconazole	− 6.69497	PHE 236, PHE 134, VAL 311, TYR 140, ILE 139, LEU 307, LEU 147, ILE 471, CYS 470, VAL 154	THR 130, GLN 150, HIS 468	LYS 151 (H-bond with triazole)	ARG 469 (charged positive), GLY 314, GLY 310, LYS 151 (Pi-cation with aromatic ring), PHE 134 (Pi-Pi stacking with triazole)

**Fig. 4:**
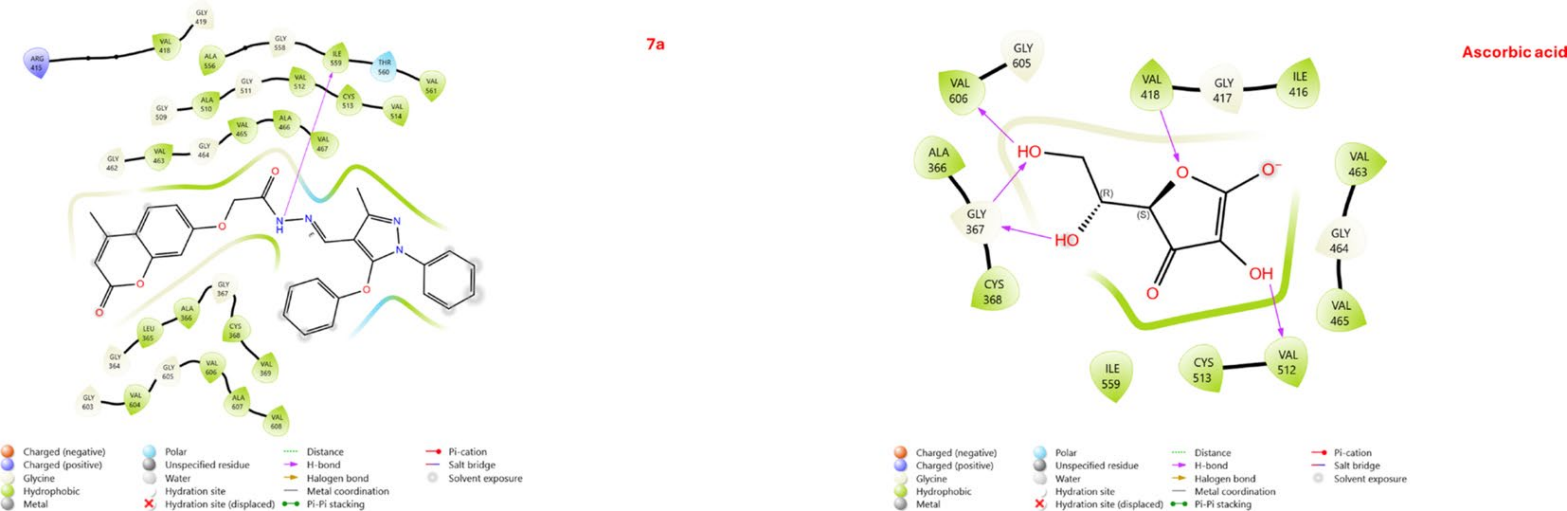
2D Molecular docking interactions of compound **7a** and standard drug ascorbic acid with the 5FNR protein target.

Compound **7a** exhibited a binding energy of − 8.9 kcal/mol against 5FNR, characterized by the absence of hydrogen bonds and the presence of hydrophobic interactions with residues VAL A:463, ALA A:510, VAL A:465, VAL A:467, LEU A:468, CYS A:513, ILE A:559, LEU A:557, ALA A:556, VAL A:561, ALA A:366, LEU A:365, VAL A:369, CYS A:368, VAL A:420, VAL A:418, VAL A:604, ALA A:607, and VAL A:608. This binding energy surpasses that of the standard drug ascorbic acid, which binds 5FNR with a binding energy of − 5.0 kcal/mol. Ascorbic acid forms hydrogen bonds with VAL A:606, GLY A:367, VAL A:512, and VAL A:418, in addition to hydrophobic interactions with ALA A:366, CYS A:368, ILE A:559, CYS A:513, VAL A:465, VAL A:463, and ILE A:416 (Fig. 4; Table 7).

**Table 7:** Binding interaction of compound **7a** with (PDB ID:5FNR).

Interacting amino acids					
Compound code	Docking score (kcal/mol)	Hydrophobic interactions	Polar interactions	H-bond	Others
**7a**	− 8.95446	VAL 418, ALA 556, ALA 510, VAL 463, VAL 465, ALA 466, VAL 512, CYS 513, VAL 467, VAL 514, VAL 561, VAL 608, ALA 607, VAL 369, CYS 368, ALA 366, LEU 365, VAL 604, VAL 467	THR 560	–	ARG 415 (charged positive), GLY 462, GLY 509, GLY 464, GLY 511, GLY 558, GLY 367, GLY 605, GLY 364, GLY 603, GLY 419
Ascorbic acid	− 5.05538	CYS 368, ALA 366, ILE 416, VAL 463, VAL 465, CYS 513 ILE 559	–	VAL 512, VAL 418, VAL 606, GLY 367 (H-bond with cyclic lactone and 3 OH)	GLY 605, GLY 417, GLY 464

Compounds **7a** and **7b** demonstrate robust and stable predicted interactions with their respective receptors, with calculated docking scores superior to those of the respective reference ligands. It must be emphasised, however, that molecular docking provides only a computational prediction of binding affinity and does not confirm biological superiority over standard drugs; experimental validation remains essential. Comprehensive docking data are presented in Tables 5, 6, and 7.

### DFT calculation

#### Molecular geometry

**Fig. 5:**
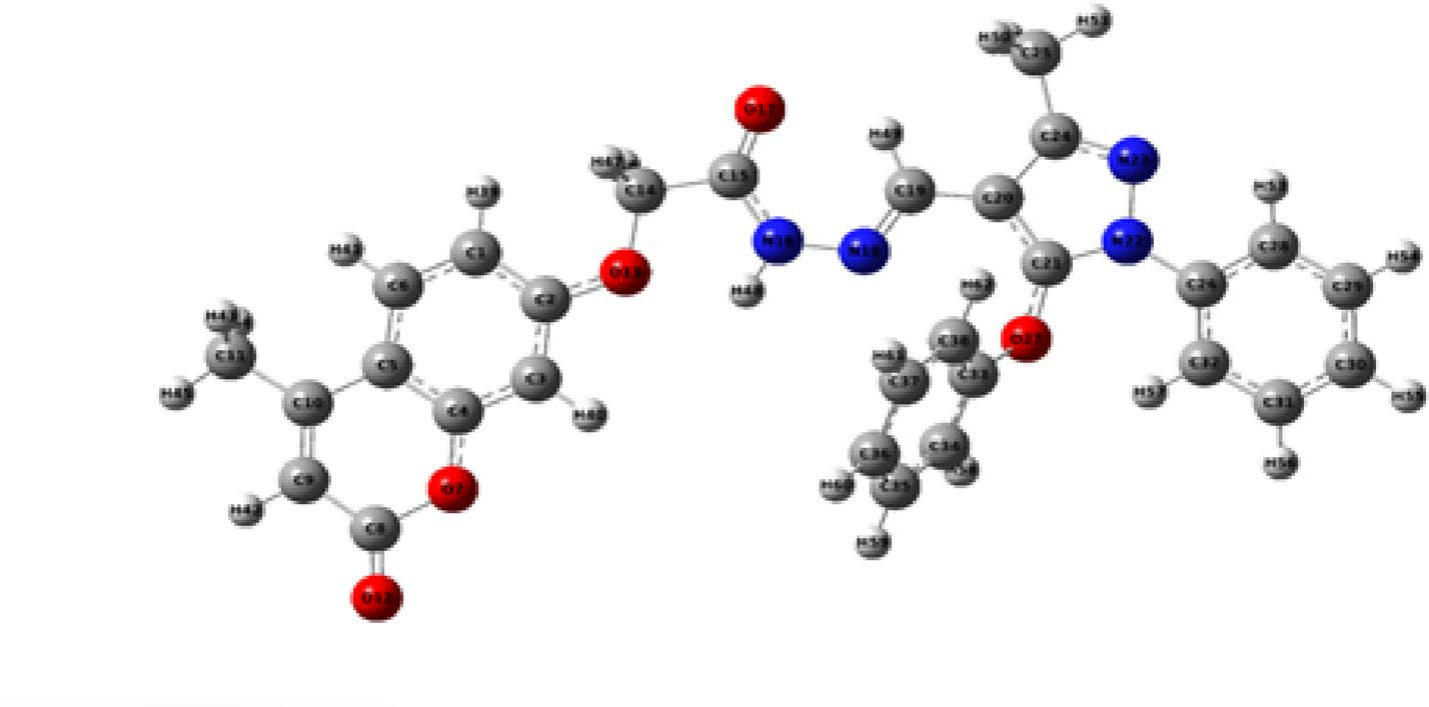
DFT-optimized molecular geometry of compound **7a** obtained at the 6–31++ G(d,p) level of theory, showing the lowest-energy conformation with atom labeling.

Density functional theory (DFT) calculations were used to obtain the molecular geometry by complete optimization of all atomic positions to locate the global minimum on the potential energy surface using the 6–31++ G(d,p) basis set. The optimization procedure is based on accurate evaluation of the electron density, ensuring convergence to the most stable molecular structure. The resulting optimized geometry represents the energetically most favorable conformation of the molecule within the chosen exchange–correlation functional and basis set. The optimized structure of compound **7a**, along with the corresponding atomic labeling, is depicted in Fig. 5^23^.

#### Mulliken population analysis

**Fig. 6:**
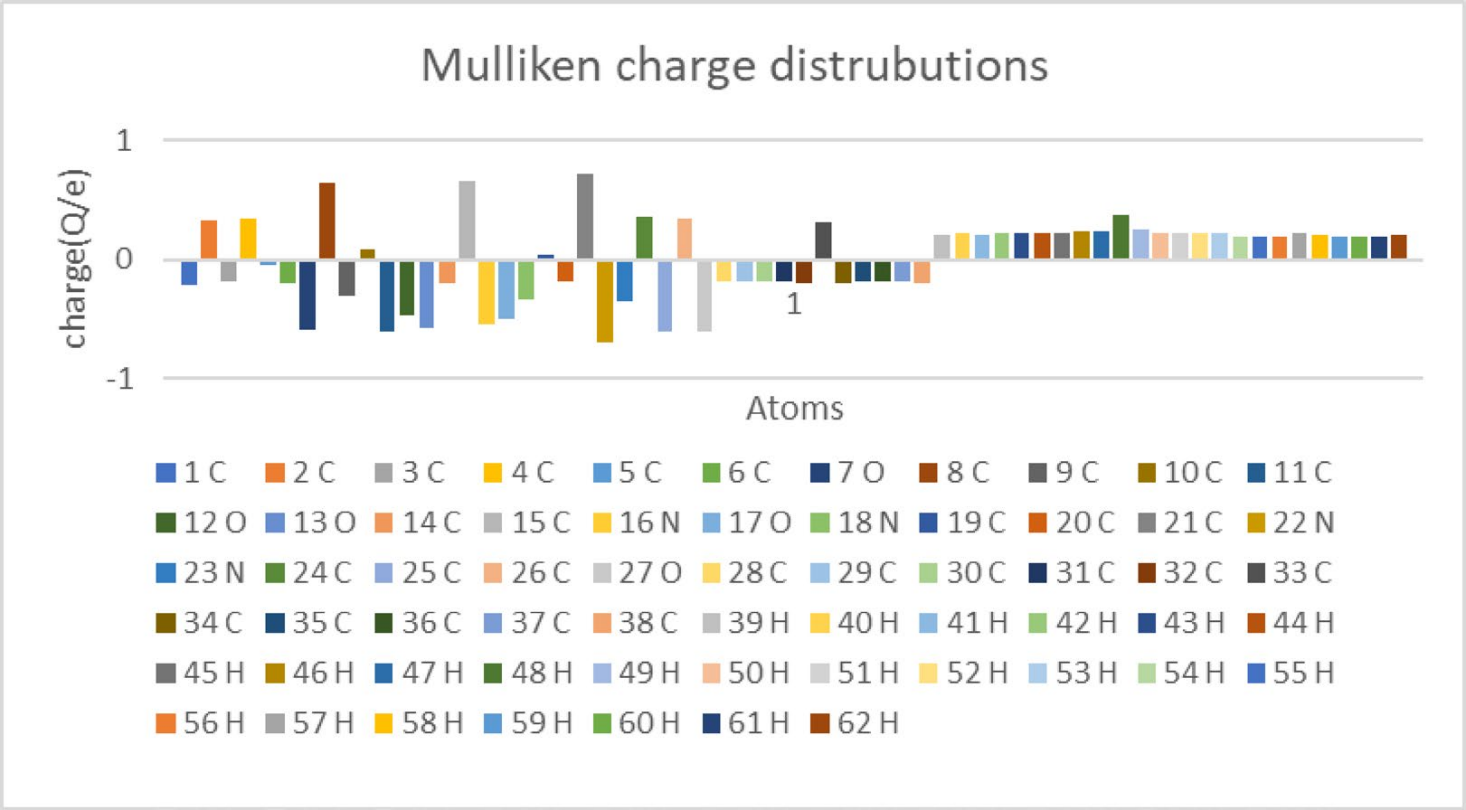
Mulliken charge distribution of compound **7a** calculated using DFT/B3LYP/6–31++ G(d,p) basis set.

The Mulliken charge distribution analysis of compound **7a** reveals a highly polarized molecular structure with significant charge separation that fundamentally governs its reactivity and interaction behavior. The molecule exhibits charges ranging from − 0.703 to + 0.717 eV, indicating substantial electronic redistribution across different functional groups. The nitrogen atoms demonstrate the most substantial electron accumulation, with N22 being the most electron-rich center at − 0.703 eV, followed by N16 (− 0.550 eV), N23 (− 0.345 eV), and N18 (− 0.329 eV), positioning these sites as prime nucleophilic centers and hydrogen bond acceptors. Similarly, the oxygen atoms consistently bear negative charges, with O27 (− 0.606 eV), C11 (− 0.605 eV), and O7 (− 0.589 eV) showing powerful electron-donating character, likely corresponding to carbonyl or hydroxyl functionalities. In contrast, the carbon framework displays significant electron deficiency at specific sites, most notably C21 (+ 0.717 eV), which represents the most electrophilic center in the molecule, along with C15 (+ 0.651 eV) and C8 (+ 0.649 eV), all of which are characteristic of highly polarized carbonyl carbons adjacent to electron-withdrawing groups. The hydrogen atoms uniformly carry positive charges ranging from + 0.19 to + 0.37 eV, with H48 (+ 0.368 eV) indicating an acidic N–H or O–H proton capable of hydrogen bond donation. This polarised charge landscape rationalises the multi-site binding interactions observed in docking studies and supports the compound’s observed hydrogen-bonding capacity with active-site residues. The Mulliken analysis thus provides electronic-level support for compound **7a**’s reactivity and binding behaviour in biological contexts (Fig. 6). **7a’s** electronic structure, revealing that its reactivity profile, binding affinity, polarizability, and potential biological activity are directly influenced by these strategically distributed electron-rich and electron-deficient centers throughout the molecular framework (Fig. 6).

#### Molecular electrostatic potential (MEP)

**Fig. 7:**
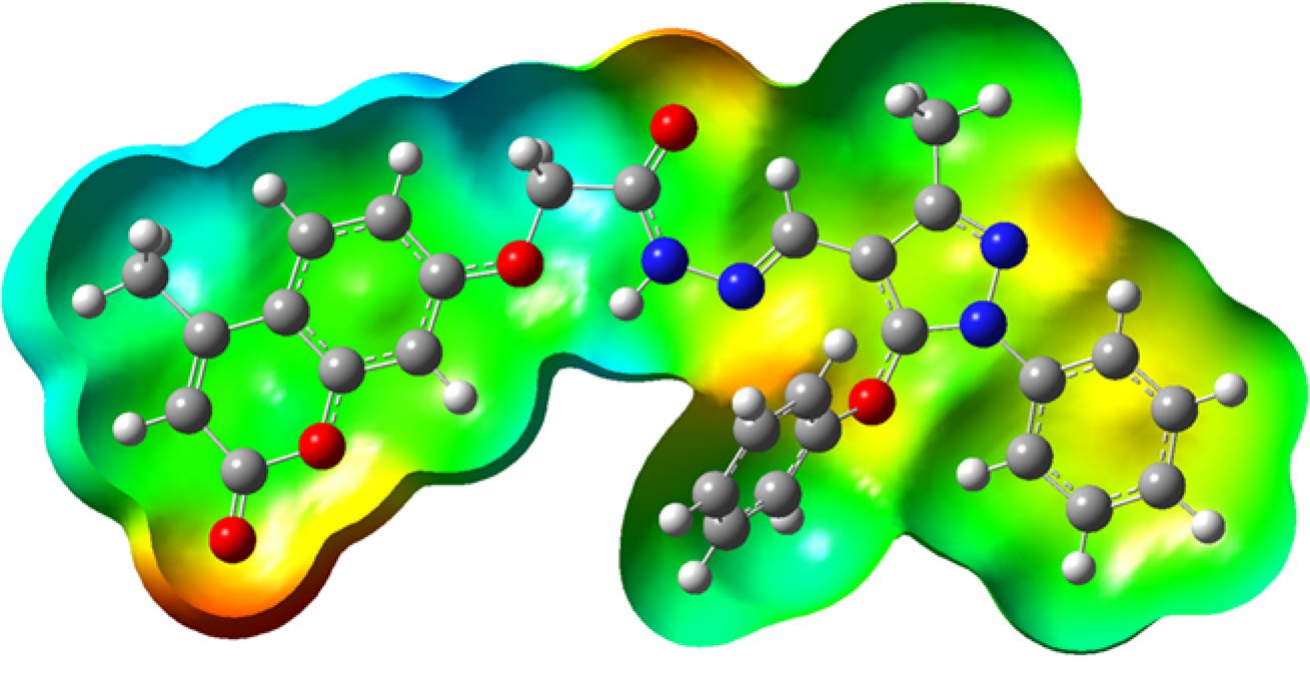
Molecular electrostatic potential (MEP) surface of compound **7a** calculated using DFT/B3LYP/6–31++ G(d,p) basis set.

The molecular electrostatic potential (MEP) surface of compound **7a**, computed at the DFT/B3LYP/6–31++ G(d,p) level, provides crucial insights into its reactivity patterns and interaction behavior with biological targets. The three-dimensional MEP map (Fig. 7) displays a color gradient where red regions represent negative electrostatic potential (electron-rich sites), blue regions indicate positive potential (electron-deficient sites), and green areas correspond to neutral zones. The analysis reveals that oxygen atoms, particularly those in carbonyl and hydroxyl groups, exhibit intense red coloration, confirming their role as strong electron donors and preferred sites for electrophilic attack and hydrogen bond acceptance. Conversely, the hydrogen atoms, especially those attached to nitrogen and oxygen, display blue regions indicative of positive potential, identifying them as hydrogen bond donors and sites favorable for nucleophilic interactions. The aromatic rings show intermediate green-to-yellow coloration, reflecting delocalized π-electron density that facilitates π–π stacking interactions. This electrostatic distribution directly correlates with the Mulliken charge analysis. It explains the compound’s capacity for molecular recognition, non-covalent binding, and orientation during biomolecular interactions, ultimately governing its pharmacological activity and binding affinity toward target receptors^24^.

#### Frontier molecular orbitals (FMOs)

**Fig. 8:**
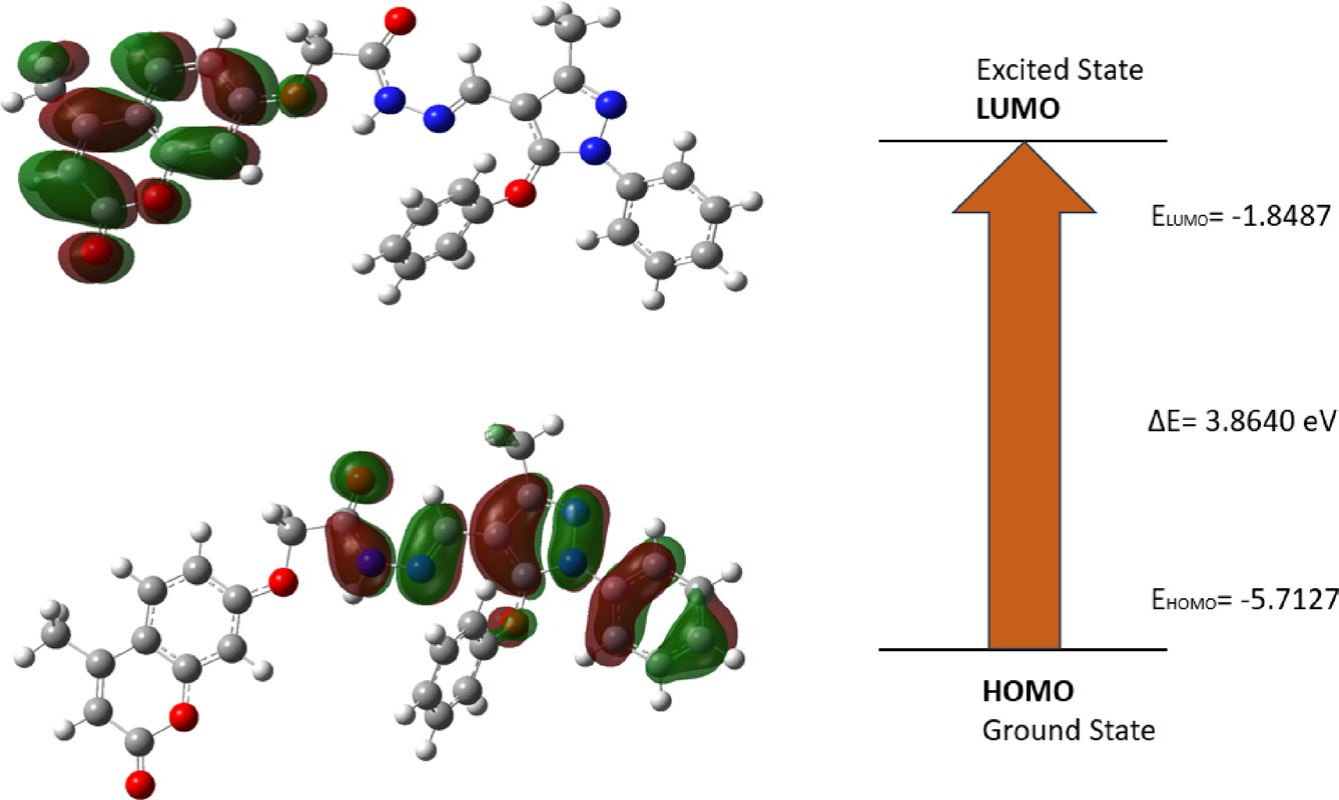
Frontier molecular orbitals and energy diagram of compound **7a** calculated using DFT/B3LYP/6–31++ G(d,p).

Frontier molecular orbital (FMO) analysis of compound **7a** was performed using DFT at the B3LYP/6–31++ G(d,p) level to evaluate its electronic properties and chemical reactivity. The HOMO, positioned at − 5.7127 eV, represents the electron-donating ability and is predominantly localized on electron-rich aromatic rings and heteroatoms (oxygen and nitrogen), identifying these regions as primary sites for electrophilic attack and oxidation. The LUMO, located at − 1.8487 eV, reflects the electron-accepting capacity and shows significant distribution over electron-deficient carbonyl carbons and conjugated systems, marking preferred sites for nucleophilic attack and reduction. The calculated HOMO–LUMO energy gap (ΔE) of 3.8640 eV serves as a key indicator of the molecule’s kinetic stability, chemical hardness, and reactivity profile. This moderate energy gap suggests that compound **7a** maintains reasonable thermodynamic stability while retaining sufficient reactivity for potential biological interactions and redox processes. Furthermore, this energy difference directly correlates with the compound’s optical properties, governing electronic transitions and UV–visible absorption characteristics, which are crucial for understanding its photophysical behavior and potential pharmacological applications (Fig. 8).

#### Global reactivity descriptors

Global reactivity descriptors derived from frontier molecular orbital theory provide a comprehensive understanding of the intrinsic chemical behavior and stability of compound **7a**. The key descriptors (Table 8) are discussed in relation to the observed biological activity. The HOMO–LUMO gap (3.8640 eV) indicates moderate kinetic stability combined with sufficient reactivity to engage biological targets, consistent with the broad-spectrum antimicrobial and antioxidant activity observed experimentally. The moderate electrophilicity index (ω = 3.6991 eV) and chemical hardness (η = 1.9320 eV) indicate that compound **7a** can engage in both nucleophilic and electrophilic interactions with receptor residues, supporting its multi-target binding profile seen in docking studies. The dipole moment of 5.1940 Debye confirms significant molecular polarity, which correlates with the compound’s solubility in polar solvents and capacity for electrostatic interactions with charged active-site residues^25^.

**Table 8:** Quantum chemical descriptors of **7a**.

Global reactivity parameters	DFT/B3LYP/6–311++ G(d,p)
E_*HOMO*_	− 5.7127 eV
E_*LUMO*_	− 1.8487 eV
Electronegativity (v)	3.7807 eV
Hardness (g)	1.9320 eV
Chemical potential (µ)	− 3.7807 eV
Ionization potential (I)	5.7127 eV
Electron affinity (A)	1.8487 eV
Energy gap (Eg)	3.8640 eV
Electrophilicity index (x)	3.6991 eV
Chemical softness (S)	0.5175 eV^−1^
Dipole moment	Debye

#### Density of states

**Fig. 9:**
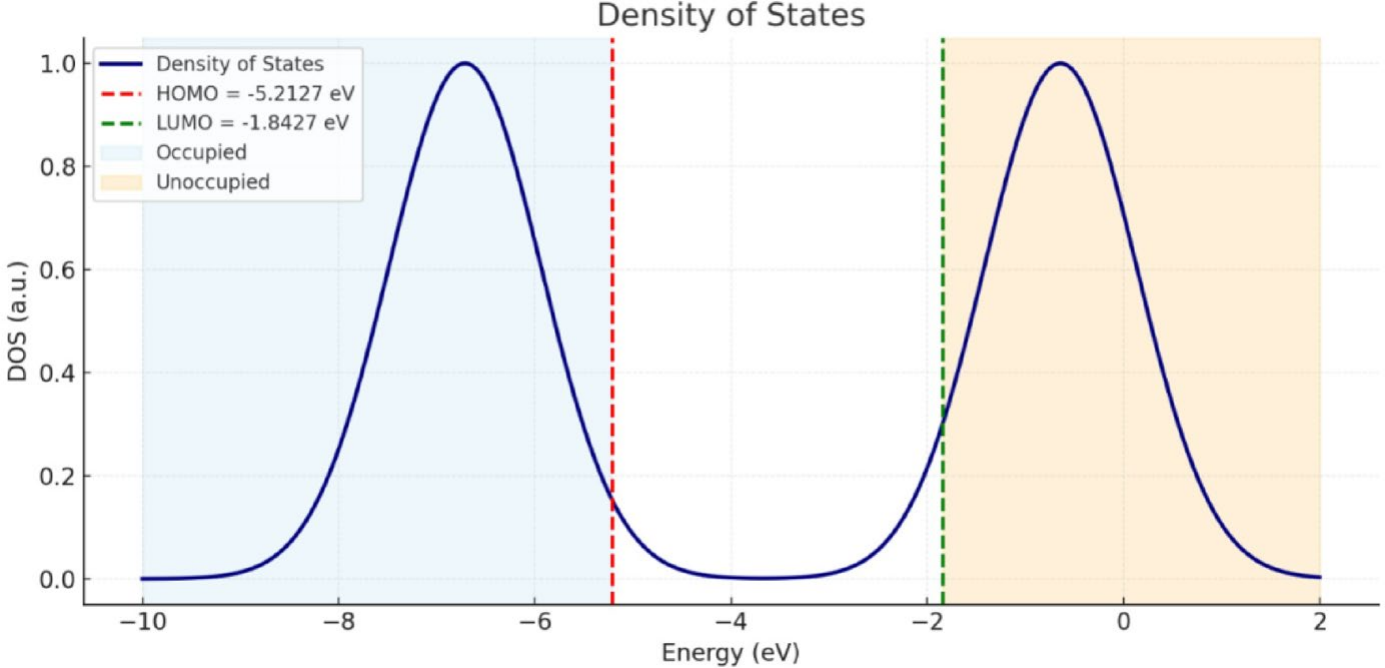
Density of states (DOS) spectrum of compound **7a** calculated using DFT/B3LYP/6–31++ G(d,p).

The density of states (DOS) analysis provides a detailed representation of the electronic structure and energy distribution of molecular orbitals in compound **7a**, complementing the frontier molecular orbital analysis. The DOS spectrum, calculated at B3LYP/6–31++ G(d,p), shows a clear separation between occupied states (below HOMO, − 5.2127 eV) and unoccupied states (above LUMO, − 1.8427 eV), yielding a gap of 3.8640 eV consistent with the HOMO–LUMO analysis. The broad distribution of occupied states reflects extensive π-electron delocalisation across the conjugated framework, thereby supporting charge-transfer capacity and correlating with the observed electronic transitions in UV–visible absorption data. This DOS profile thus validates the calculated electronic structure and corroborates the reactivity descriptors discussed in “Global reactivity descriptors”^26^ (Fig. 9).

#### Thermodynamic properties and energy partitioning

The thermodynamic parameters of compound **7a**, calculated using DFT at the B3LYP/6–31++ G(d,p) level, are summarised in Table 9. The zero-point vibrational energy (ZPVE = 304.1963 kcal/mol) provides the baseline energy correction for thermochemical calculations. The total internal energy at standard conditions is 324.549 kcal/mol, of which vibrational contributions dominate (322.771 kcal/mol, 99.5%), as expected for a molecule of this size and conformational complexity. These parameters confirm molecular stability under ambient conditions and are relevant to understanding the compound’s thermochemical behaviour in biological environments.

**Table 9:** Calculated thermodynamic parameters of compound **7a** at the B3LYP/6–31++ G(d,p) level.

Sr. No.	Parameters	B3LYP 6–311++ G (d, p)
1	Zero-point vibrational energy (kcal/mol)	304.1963 (Kcal/Mol)
2	Rotational temperature (K)	0.00996 0.00162 0.00144
3	Rotational constant (GHz)	X = 0.20747 Y = 0.03381 Z = 0.03008
4	Total energy E total (kcal/mol)	324.549
5	Translational (kcal/mol)	0.889
6	Rotational (kcal/mol)	0.889
7	Vibrational (kcal/mol)	322.771

#### Non-covalent interaction (NCI) analysis via reduced density gradient (RDG)

**Fig. 10:**
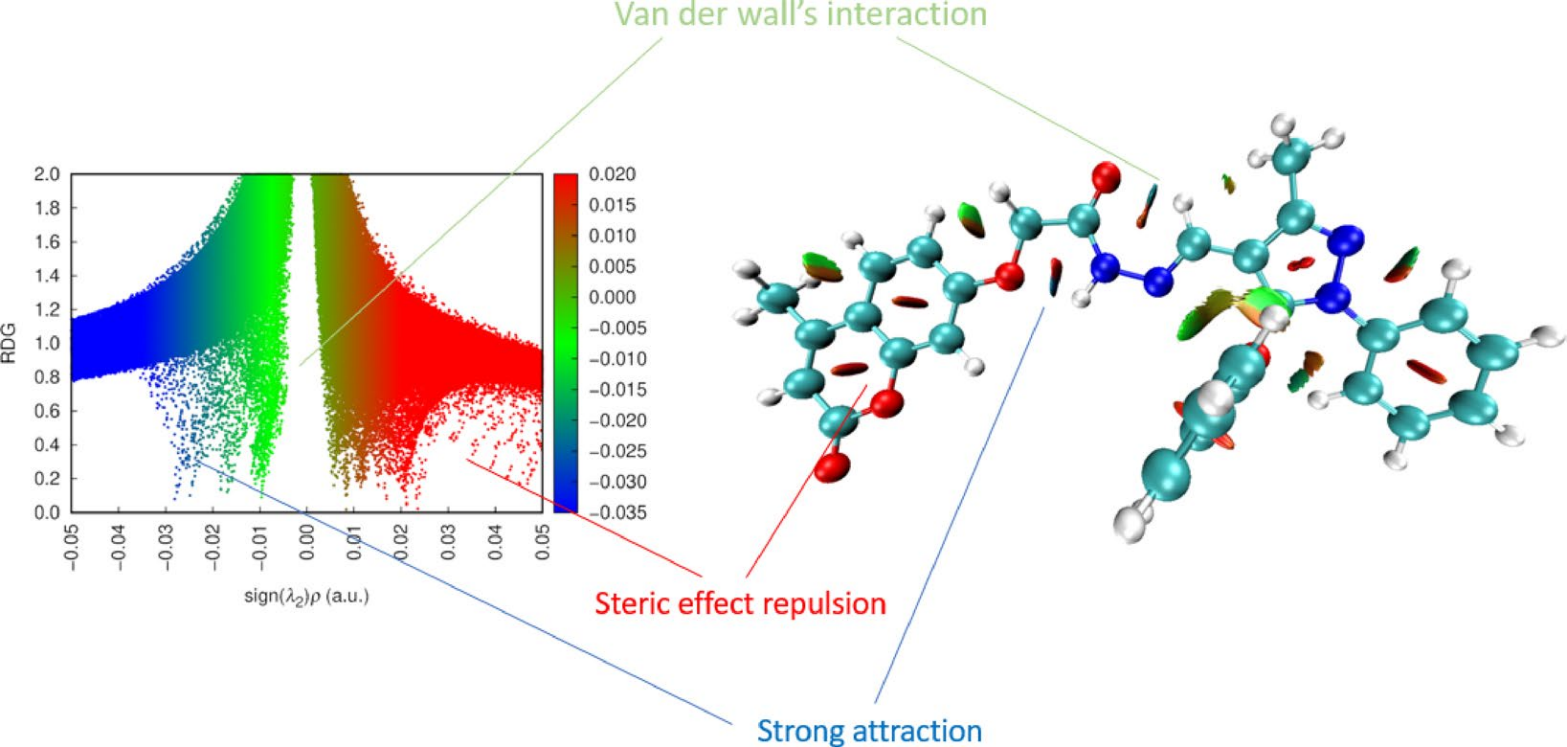
Non-covalent interaction (NCI) analysis of compound **7a** using reduced density gradient (RDG) method.

The reduced density gradient (RDG) analysis provides a comprehensive visualization of non-covalent interactions in compound **7a**, revealing the nature and strength of weak intermolecular and intramolecular forces that govern its molecular stability and binding behavior. Developed by Johnson et al., the RDG method employs a dimensionless parameter derived from electron density (ρ) and its gradient to identify regions of non-covalent interactions. The RDG scatter plot (Fig. 13, left) displays sign(λ_2_)ρ values on the x-axis against RDG values on the y-axis, with color coding indicating interaction types: blue regions correspond to strong attractive interactions such as hydrogen bonding (negative sign(λ_2_)ρ values), green regions represent weak van der Waals interactions (sign(λ_2_)ρ near zero), and red regions indicate steric repulsion or unfavorable overlaps (positive sign(λ_2_)ρ values). The three-dimensional NCI isosurface visualization (Fig. 10) spatially maps these interactions onto the molecular structure using the same color scheme. Analysis of compound **7a** reveals several key interaction features: pronounced steric repulsion (red isosurfaces) between the Coumarin, Pyrazole, and phenyl rings due to spatial crowding; van der Waals interactions (green isosurfaces) between the Pyrazole and phenyl fragments, particularly involving C–H···π contacts; strong attractive interactions (blue isosurfaces) between the methyl substituent and the coumarin ring system; and dual-character interactions at the nitro groups showing both van der Waals attractions and steric effects. These non-covalent interactions collectively determine the preferred conformational geometry, intramolecular stability, and intermolecular recognition properties of compound **7a**, providing crucial insights for understanding its binding affinity with biological targets and rational drug design optimization.

#### Electron localization function (ELF) and localized orbital locator (LOL) analysis

**Fig. 11:**
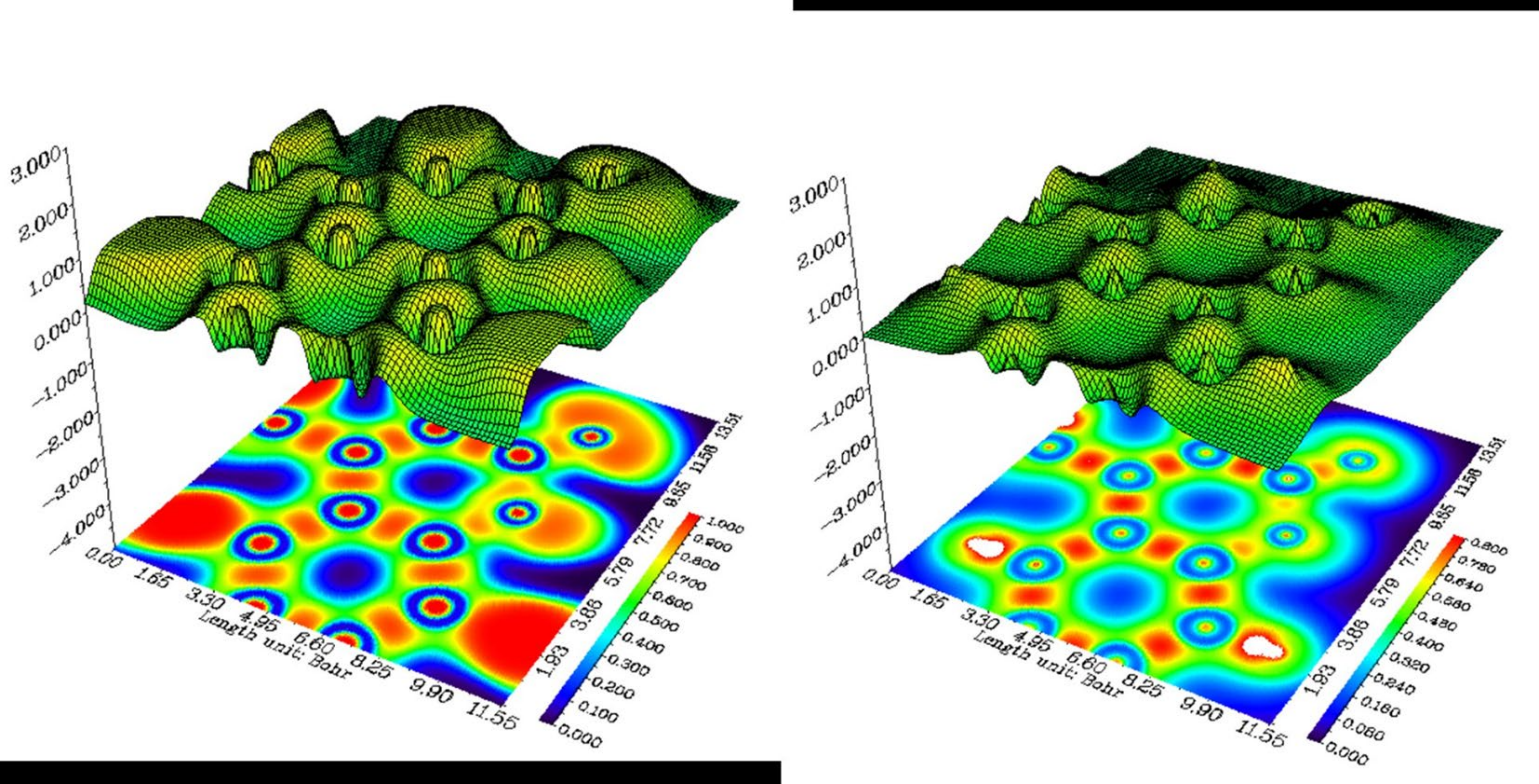
Electron localization function (ELF) and localized orbital locator (LOL) maps of compound **7a**.

The electron localization function (ELF) and localized orbital locator (LOL) analyses provide complementary insights into the spatial distribution of electron pairs and bonding characteristics in compound **7a**. Both descriptors were computed using Multiwfn software and visualized as three-dimensional isosurfaces maps with two-dimensional contour projections (Fig. 11). ELF quantifies the likelihood of finding electron pairs in specific molecular regions, with values ranging from 0.0 (complete delocalization) to 1.0 (maximum localization), while LOL measures electron localization based on kinetic energy density, with values spanning 0.0 to 0.8. The color-coded maps display red regions indicating high electron localization (ELF/LOL > 0.8), corresponding to covalent bond centers, lone pair domains, and atomic cores. In contrast, blue regions represent electron-depleted zones (ELF/LOL < 0.3) associated with interatomic boundaries and valence-shell depletion. In compound **7a**, the highest localization values (0.8–1.0) are observed at covalent bond critical points, particularly C–C, C–N, C–O, and C–H bonds, reflecting strong electron-pair sharing and localized bonding interactions. The oxygen and nitrogen heteroatoms exhibit pronounced ELF/LOL maxima due to localized lone pairs, which are crucial for hydrogen bonding and electrophilic attack. Circular blue zones surrounding atomic nuclei indicate core electron regions, while green-to-yellow transition zones represent bonding electron density between adjacent atoms. Notably, the aromatic rings display moderate localization values (0.5–0.7), reflecting π-electron delocalization across the conjugated system. At the same time, the pyrazole and coumarin moieties show distinct localization patterns influenced by nitrogen and oxygen substituents. The hydrogen atoms connected to nitrogen and oxygen exhibit higher ELF values, confirming their participation in polarized bonds and potential hydrogen bonding interactions. These complementary ELF and LOL maps reveal how substituents, particularly nitrogen, oxygen, and carbonyl groups, modulate electron distribution, influence molecular reactivity, and determine the preferred sites for intermolecular interactions in compound** 7a**.

### ADME and toxicity profile analysis

#### In silico ADME-Tox evaluation

The pharmacokinetic and toxicological properties of synthesized compounds **7a–e** and **11a–e** were comprehensively evaluated using SwissADME, ADMETlab 3.0, and ProTox 3.0 platforms to assess their drug-likeness and safety profiles.

#### Physicochemical and drug-likeness properties

The molecular weights of the compounds ranged from 479.48 to 558.58 Da, with **7a–e** generally exhibiting higher molecular weights than **11a–e**. Most compounds demonstrated acceptable lipophilicity, with Csp3 fraction values between 0.07 and 0.13, indicating appropriate saturation for favourable drug properties. The number of rotatable bonds (nROTB) ranged from 8 to 10, suggesting moderate molecular flexibility. All compounds possessed 7–9 hydrogen-bond acceptors and predominantly 1 hydrogen-bond donor, with topological polar surface areas (TPSA) ranging from 103.02 to 153.77 Å^2^, which fall within the acceptable range for oral bioavailability.

Drug-likeness assessment revealed that compounds **7a–d** and **11b–d** exhibited 0–2 violations across Lipinski, Ghose, Veber, Egan, and Muegge rules, indicating favorable drug-like characteristics. Notably, compounds **7e** and **11e** each had 2 Lipinski violations and bioavailability scores of 0.17, primarily due to increased molecular complexity and TPSA values exceeding 140 Ų. The bioavailability scores for most compounds ranged from 0.55, suggesting moderate to good oral bioavailability potential, with compounds **7a–d** and **11a–d** demonstrating superior profiles.

#### Absorption and distribution parameters

Caco-2 permeability predictions indicated moderate intestinal absorption potential across all compounds, with values ranging from −5.42 to − 5.25 log units. The MDCK cell permeability values (− 4.85 to − 4.68) indicated acceptable membrane penetration. P-glycoprotein (Pgp) substrate predictions showed generally low probabilities (0.001–0.049), indicating minimal efflux liability, which is favorable for maintaining intracellular drug concentrations. The volume of distribution at steady state (VDss) values were negative (− 0.39 to − 0.15), suggesting predominantly plasma protein binding with limited tissue distribution.

Blood–brain barrier (BBB) permeability was extremely low (10^−8^ to 10^−6^) for all compounds, indicating minimal CNS penetration, which is advantageous for peripherally acting drugs and reduces the risk of CNS-related side effects. OATP1B1 and OATP1B3 transporter interaction probabilities were high (0.70–0.99), suggesting potential hepatic uptake and possible involvement in hepatobiliary elimination pathways.

#### Metabolic stability profile

Cytochrome P450 enzyme interaction predictions revealed favorable metabolic profiles. All compounds showed low inhibition potential for CYP1A2, CYP2C19, CYP2C9, and CYP2D6 isoforms, with probabilities exceeding 0.60 for non-inhibition. CYP3A4 substrate probabilities were minimal (0.0001–0.02), suggesting alternative metabolic pathways or metabolic stability. The CYP2C8 inhibition probabilities varied considerably, with compounds **7a–e** showing higher probabilities (0.66–0.99) compared to **11a–e** series. This differential metabolism profile indicates that structural modifications between the two series significantly influence metabolic liability.

#### Excretion and pharmacokinetic parameters

Plasma clearance (CL-plasma) values ranged from 1.76 to 2.61 mL/min/kg, indicating moderate elimination rates. The predicted half-life (T1/2) values clustered around 0.94–1.08, suggesting a reasonable duration of action suitable for therapeutic applications. These parameters collectively indicate that the compounds would require moderate dosing frequencies to maintain therapeutic concentrations.

#### Toxicity risk assessment

Ames mutagenicity predictions indicated a low-to-moderate risk, with probabilities ranging from 0.77 to 0.97, suggesting potential genotoxicity requiring experimental validation. The hERG-I and hERG-II cardiotoxicity predictions indicated moderate risk (0.51–0.64 and 0.51–0.59, respectively), suggesting the potential for QT interval prolongation and warranting cardiac safety evaluation in preclinical studies.

**Fig. 12:**
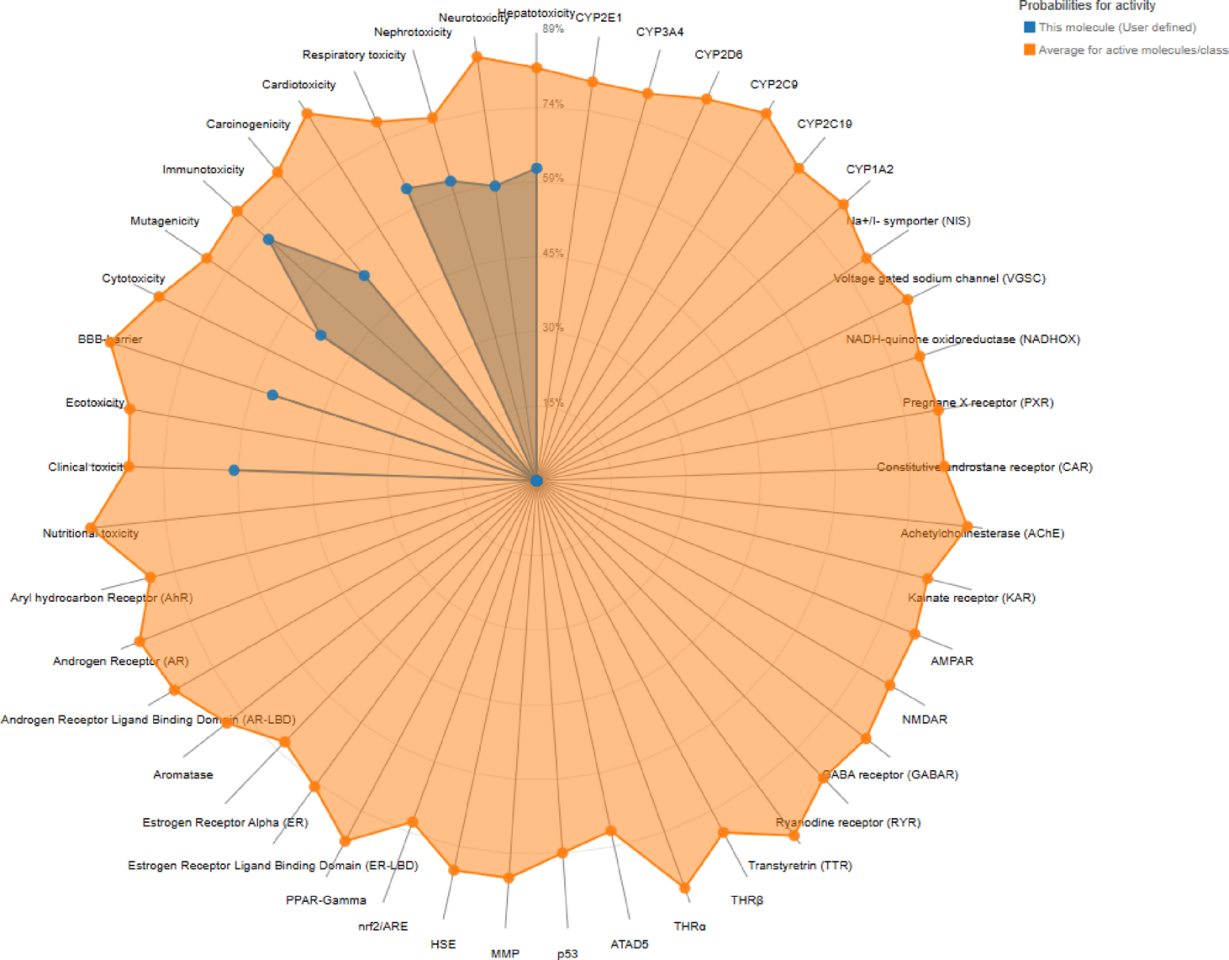
Multi-target activity profile and toxicity prediction of compound **11a**.

**Fig. 13:**
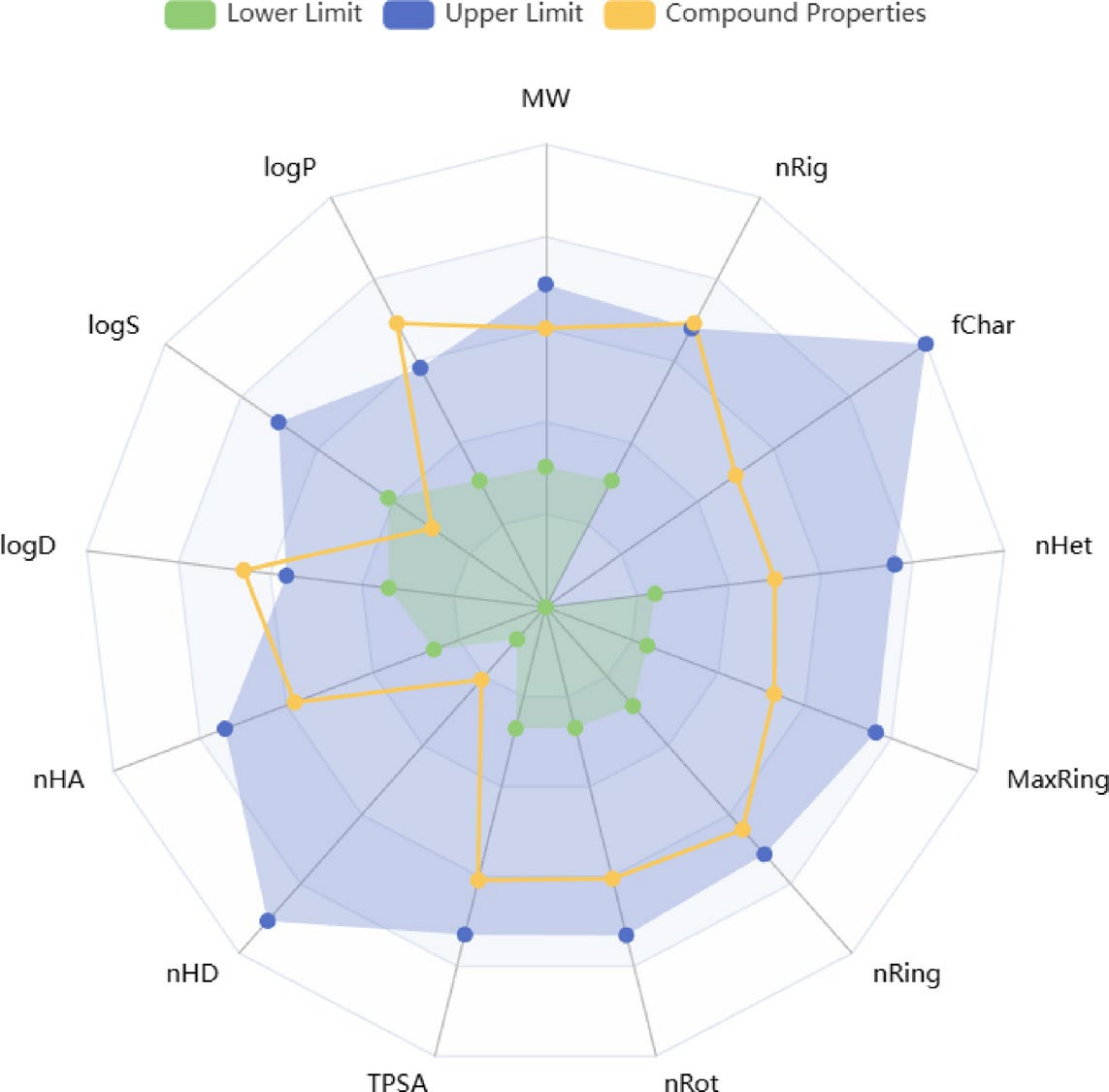
Physicochemical property radar plot for compound **11a** against drug-likeness boundaries.

**Fig. 14:**
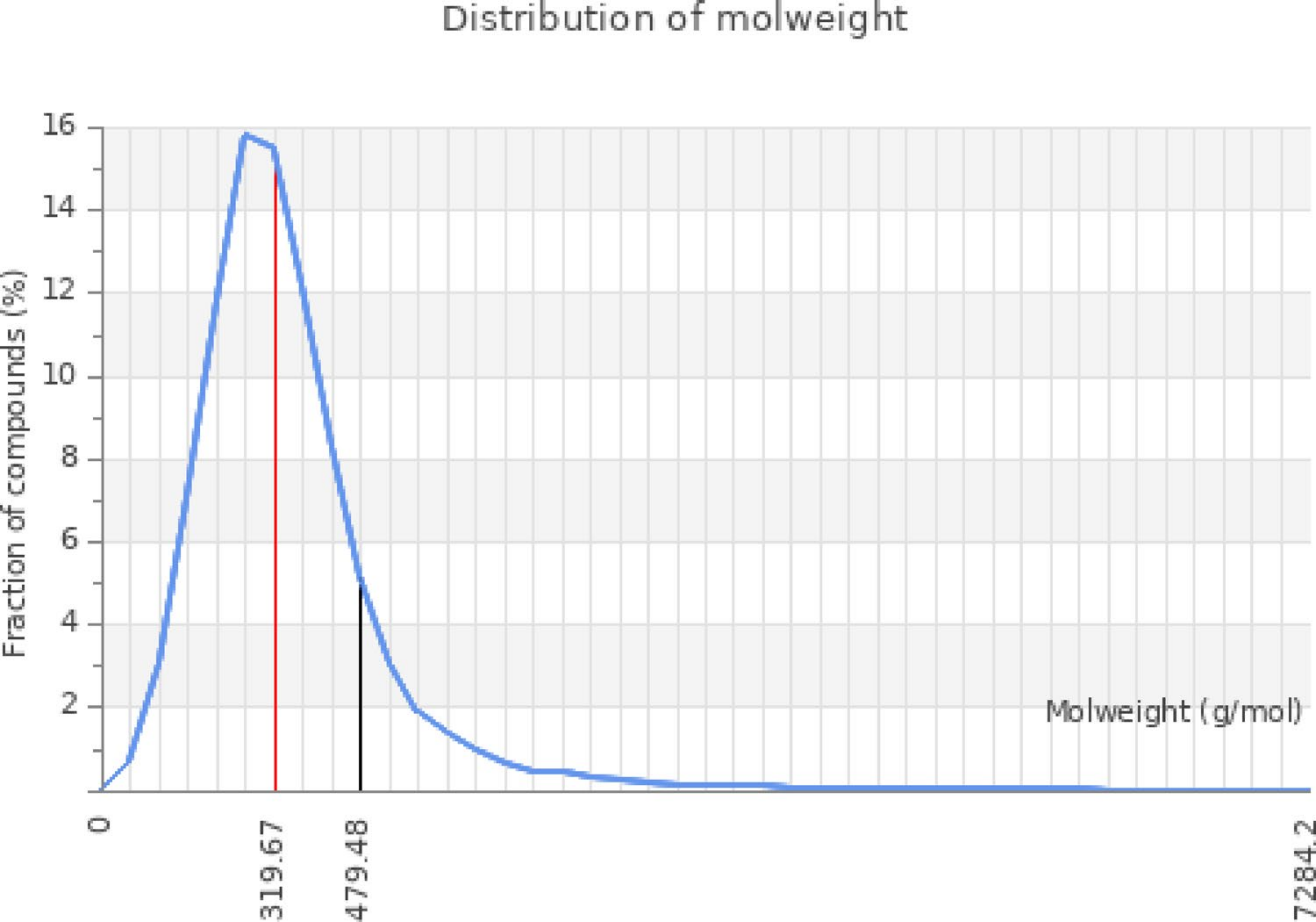
Molecular weight distribution analysis of compound **11a**.

**Fig. 15:**
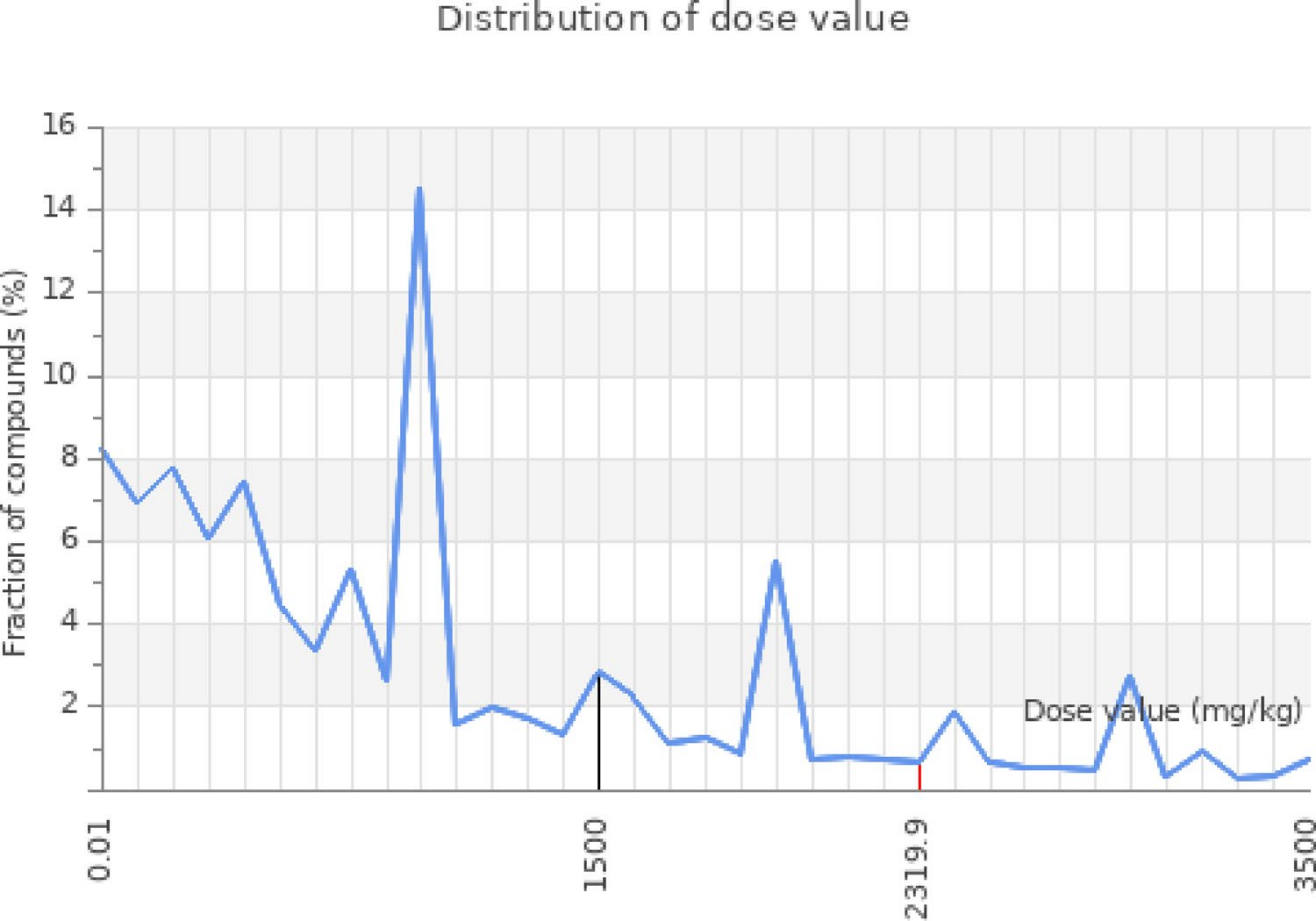
Dose value distribution profile for compound **11a**.

Skin sensitization potential varied considerably, with compounds **7e** and **11e** showing higher probabilities (0.81 and 0.74), potentially limiting topical application routes. Predictions of ototoxicity, neurotoxicity, nephrotoxicity, and hematotoxicity generally indicated low- to moderate-risk profiles across the series. Notably, compound **7e** exhibited lower neurotoxicity potential (0.07) compared to other analogs. Genotoxicity predictions were consistently high (> 0.99) across all compounds, underscoring the critical need for comprehensive in vitro and in vivo genotoxicity studies before advancing these candidates (Figs. 12, 13, 14 and 15; Tables 10 and 11).

**Table 10:** Physicochemical properties and drug-likeness assessment of synthesized derivatives **7a–e** and **11a–e**.

S. No.	Physiological properties							Drug likeness properties (#violations)					
	MW	Heavy atoms	Csp3	nROTB	HBA	HBD	TPSA	Lipinski	Ghose	Veber	Egan	Muegge	Bio. score
**7a**	508.52	38	0.1	9	7	1	107.95	1	2	0	0	1	0.55
**7b**	558.58	42	0.09	9	7	1	107.95	1	3	0	1	1	0.55
**7c**	522.55	39	0.13	9	7	1	107.95	1	2	0	0	1	0.55
**7d**	522.55	39	0.13	9	7	1	107.95	1	2	0	0	1	0.55
**7e**	553.52	41	0.1	10	9	1	153.77	2	2	1	1	1	0.17
**11a**	479.48	36	0.07	8	7	1	103.02	0	1	0	0	0	0.55
**11b**	493.51	37	0.1	8	7	1	103.02	0	2	0	0	1	0.55
**11c**	493.51	37	0.1	8	7	1	103.02	0	2	0	0	1	0.55
**11d**	493.51	37	0.1	8	7	1	103.02	0	2	0	0	1	0.55
**11e**	524.48	39	0.07	9	9	1	148.84	2	2	1	1	0	0.17

**Table 11:** In silico ADMET profiling of synthesized derivatives **7a–e** and **11a–e**: comprehensive pharmacokinetic and toxicity predictions.

Entry	**7a**	**7b**	**7c**	**7d**	**7e**	**11a**	**11b**	**11c**	**11d**	**11e**
Absorption/distribution										
CaCo2	− 5.35253	− 5.40776	− 5.41015	− 5.39046	− 5.42457	− 5.25465	− 5.3552	− 5.36144	− 5.34052	− 5.35905
MDCK	− 4.84922	− 4.84372	− 4.81011	− 4.82818	− 4.78946	− 4.68377	− 4.73611	− 4.70678	− 4.72662	− 4.71092
pgp	0.001684	0.001165	0.001618	0.003863	0.000576	0.020707	0.038163	0.036018	0.049717	0.011411
VDss	− 0.32063	− 0.1522	− 0.24355	− 0.24404	− 0.24854	− 0.38973	− 0.31681	− 0.32962	− 0.30173	− 0.32747
BBB	2.39E−06	9.91E−08	1.35E−06	1.46E−06	2.39E−08	4.63E−07	4.82E−07	2.61E−07	4.76E−07	9.26E−09
OATP1B1	0.77251	0.863718	0.962846	0.926048	0.706204	0.889045	0.977069	0.962938	0.94456	0.800062
OATP1B3	0.999727	0.999846	0.999827	0.999569	0.999875	0.999974	0.999966	0.999959	0.999978	0.999975
Metabolism										
CYP1A2-	0.725814	0.882497	0.621661	0.688292	0.386772	0.720418	0.100763	0.221262	0.503427	0.129051
CYP2C19	0.995171	0.996308	0.998227	0.998277	0.990288	0.994994	0.992031	0.996471	0.99871	0.965284
CYP2C9	0.99966	0.999612	0.999883	0.999642	0.995942	0.220089	0.220089	0.340929	0.69587	0.024762
CYP2D6	0.995171	0.996308	0.998227	0.998277	0.990288	0.994994	0.992031	0.996471	0.99871	0.965284
CYP3A4	0.000356	0.000114	0.006189	0.000511	0.005607	0.000801	0.008245	0.002621	0.000844	0.022305
CYP2B6	0.005242	0.007862	0.040949	0.007418	0.034884	0.012716	0.002102	0.000903	0.017487	0.007697
CYP2C8	0.98899	0.970663	0.999533	0.977261	0.995227	0.945968	0.905193	0.664296	0.839831	0.724981
Excretion										
cl-plasma	2.293852	1.95964	1.764845	2.425185	2.082294	2.401611	2.047202	2.611347	2.48218	2.211753
T1/2	0.967805	0.969265	1.082627	0.972506	1.016169	0.956219	1.018881	0.957704	0.940269	0.98354
Toxicity										
Ames	0.777251	0.89982	0.773606	0.770404	0.967924	0.816883	0.811018	0.803126	0.797966	0.969047
hERG-I	0.55034	0.636016	0.586006	0.529165	0.560351	0.587579	0.612664	0.57515	0.561686	0.583619
hERG-II	0.582726	0.517762	0.593162	0.548805	0.54032	0.55689	0.566941	0.532704	0.53384	0.549814
SkinSen	0.338128	0.163862	0.347557	0.345114	0.810573	0.264366	0.270729	0.264858	0.227792	0.744495
Ototoxicity	0.276136	0.412945	0.311557	0.304079	0.239576	0.251787	0.294212	0.294761	0.312079	0.212124
Neurotoxicity-DI	0.658357	0.784963	0.697595	0.6119	0.070824	0.803404	0.821469	0.769311	0.733245	0.114301
Nephrotoxicity-DI	0.429636	0.720517	0.459987	0.435338	0.457299	0.607553	0.619287	0.610038	0.596071	0.553082
Hematotoxicity	0.230886	0.342216	0.259098	0.268984	0.418467	0.42436	0.457286	0.474952	0.500617	0.562162
Genotoxicity	0.996803	0.999549	0.996168	0.996931	1	0.997524	0.996619	0.997237	0.996883	1

## Experimental

All chemical reagents and solvents employed in the present investigation were procured from commercial suppliers, including Sigma-Aldrich, Fluka, and E. Merck, and utilised as received without additional purification unless explicitly stated. Melting point determinations were conducted using a calibrated digital melting point apparatus capable of measuring temperatures up to 350 °C. Infrared spectroscopic analyses were performed on a Shimadzu FT-IR 8000 spectrometer, with samples prepared as potassium bromide (KBr) pellets. Nuclear magnetic resonance (NMR) spectroscopy was performed on a Bruker 400 MHz instrument using deuterated dimethyl sulfoxide (DMSO-d6) as the solvent and tetramethylsilane (TMS) as the reference standard for both ^1^H and ^13^C NMR. Chemical shift values (δ) are expressed in parts per million (ppm) relative to TMS, while coupling constants (J) are given in hertz (Hz). Proton signal patterns are designated as follows: singlet (s), doublet (d), triplet (t), and multiplet (m). Mass spectrometric data were acquired using a Shimadzu LC–MS-8030 system operating under electron ionization conditions at 70 eV. Reaction progress and product purity were monitored by thin-layer chromatography (TLC) on commercially available aluminium-backed silica gel plates (60G F254, 0.25 mm thickness) with visualization under UV light at 254 nm.

**Synthesis of 2-((4-methyl-2-oxo-2** ***H*** **-chromen-7-yl)oxy)acetohydrazide (3)**

A mixture of 4-methyl-7-hydroxycoumarin (**1**) (5 g, 20 mmol), ethyl chloroacetate (3 mL, 20 mmol), and K_2_CO_3_ (4.7 g, 30 mmol) in DMF was refluxed for 2 h. After completion (TLC monitoring), the mixture was poured onto crushed ice, and the resulting solid was filtered, dried, and recrystallized from ethanol, affording ethyl 2-((4-methyl-2-oxo-2*H*-chromen-7-yl) oxy)acetate (**2**). For conversion to hydrazide (**3**), hydrazine hydrate (15 mL) was added dropwise to a solution of compound (**2**) (5 g, 19 mmol) in ethanol (20 mL). The mixture was refluxed at 70 °C for 3 h, cooled, and the solid product was filtered, washed with cold water, and dried to yield 4 g (89.77%) of compound (**3**).

**White solid; Yield: 86%; m.p.: 205 °C.**^**1**^**H NMR (400 MHz, DMSO-d**_**6**_**, *δ* ppm):** 9.08 (s, 1H, NH), 7.72 (s, 1H, Ar–H), 7.05–7.07 (dd, 1H, *J* = 8.0 Hz, Ar–H), 7.01–6.94 (d, 1H, *J* = 8.0 Hz, Ar–H), 6.23 (s, 1H, Ar–H), 4.21 (s, 2H, NH_2_), 4.51 (s, 2H, CH_2_), 2.41 (s, 3H, CH_3_). ^13^C NMR (400 MHz, DMSO-d_6_, *δ* ppm): 166.3, 161.3, 160.8, 154.3, 152.7, 125.8, 112.5, 111.9, 111.0, 104.0, 66.3, 19.4. FT-IR (ATR, cm^−1^): 3250 (NH), 1742 (C=O, lactone), 1670 (CONH), 1580 (C=C aromatic). MS (m/z): 248.24. M.F.: C_12_H_12_N_2_O_4_.

**Synthesis of 3-methyl-5-phenoxy-1-phenyl-1** ***H*** **-pyrazole-4-carbaldehyde (6)**

DMF was cooled in an ice bath, and POCl_3_ was added dropwise while maintaining the temperature at 0–5 °C. Compound (**4**) (1 g, 5 mmol) was added gradually, and the reaction mixture was refluxed for 2 h. After TLC-confirmed completion, the mixture was poured onto ice, and the solid was filtered and washed with cold water to obtain 3-methyl-1-phenyl-1*H*-pyrazole-4-carbaldehyde (**5**). Next, compound (**5**) (1 g, 4 mmol) was refluxed with phenol (0.63 mL, 4 mmol) and K_2_CO_3_ (1.2 g, 9 mmol) in DMF for 3 h. The reaction mixture was poured onto ice, and the solid was filtered, washed with water, and recrystallized to yield compound (**6**).

**White solid; Yield: 88%; m.p.: 115–116 °C.**^**1**^**H NMR (400 MHz, CDCl**_**3**_**, *δ* ppm):** 9.50 (s, 1H, CHO), 7.62 (m, 2H, Ar–H), 7.58 (m, 2H, Ar–H), 7.52 (t, 2H, Ar–H), 7.09 (t, 2H, Ar–H), 6.78–6.82 (d, 2H, *J* = 8 Hz, Ar–H), 2.60 (s, 3H, CH_3_).^13^C NMR (400 MHz, CDCl_3_, *δ* ppm): 191.0, 155.2, 149.2, 137.5, 129.7, 129.3, 126.2, 124.6, 122.7, 122.5, 114.6, 14.2. FT-IR (ATR, cm^−1^): 3094 (aromatic C–H), 2950 (aliphatic C–H), 1745 (CHO), 1275 (C–O ether), 1505 (C=C aromatic). MS (m/z): 278. M.F.: C_17_H_14_N_2_O_2_.

**Synthesis of 2-phenoxyquinoline-3-carbaldehyde (10)**

DMF was cooled in an ice bath and POCl_3_ was added dropwise while maintaining a temperature of 0–5 °C. Compound (**8**) (1 g, 7.3 mmol) was added portionwise, and the mixture was refluxed for 2 h. After the reaction was complete (TLC), the mass was poured into ice, and the solid was filtered and washed to give 2-chloroquinoline-3-carbaldehyde (**9**).

Compound (**9**) (1 g, 5 mmol) was then refluxed with phenol (0.49 mL, 5 mmol) and K_2_CO_3_ (1.4 g, 9 mmol) in DMF for 3 h. The reaction mixture was poured onto ice, and the solid was filtered, washed, and recrystallized to obtain the final product (**10**).

**White solid; Yield: 84%; m.p.: 152 °C.**^**1**^**H NMR (400 MHz, CDCl**_**3**_**, *δ* ppm):** 9.72 (s, 1H, CHO), 8.0–7.99 (d, 2H, *J* = 4 Hz, Ar–H), 7.75–7.77 (dd, 2H, *J* = 8 Hz, Ar–H), 7.53 (m, 2H, Ar–H), 7.12 (t, 2H, Ar–H), 6.86 (t, 1H, Ar–H), 6.84–6.85 (dd, 1H, *J* = 4 Hz, Ar–H). ^13^C NMR (400 MHz, CDCl_3_, *δ* ppm): 191.0, 173.7, 155.2, 145.0, 143.8, 133.3, 129.9, 129.7, 126.9, 126.3, 125.5, 124.6, 122.7, 120.4. FT-IR (ATR, cm^−1^): 2775 (aromatic C–H), 1685 (C=O), 1505 (C=C/C=N), 1205 (C–O ether). MS (m/z): 249. M.F.: C_16_H_11_N_2_O_2_.

**Synthesis of (** ***E*** **)-2-((4-methyl-2-oxo-2** ***H*** **-chromen-7-yl)oxy)-** ***N*** **-((3-methyl-5-phenoxy-1-phenyl-1** ***H*** **-pyrazol-4-yl)methylene)acetohydrazide (7a–e)**

A mixture of compound (**3**) (0.2 g, 1 mmol) and compound (**6**) (0.3 g, 1 mmol) in a 1:1 mixture of DMF and ethanol was heated under reflux for 2 h with 2–3 drops of concentrated sulfuric acid. The resulting product (7a–e) was obtained in 75% yield.

**Synthesis of (** ***E*** **)-2-((4-methyl-2-oxo-2** ***H*** **-chromen-7-yl)oxy)-** ***N*** **-((2-phenoxyquinolin-3-yl) methylene)acetohydrazide (11a–e)**

Compound (**3**) (0.1 g, 0.4 mmol) and compound (**10**) (0.1 g, 0.4 mmol) were refluxed for 2 h in a 1:1 mixture of DMF and ethanol with 2–3 drops of concentrated sulfuric acid to yield compound (**11a–e**) in 78% yield.

### Spectral analysis

#### (*E*)-2-((4-methyl-2-oxo-2H-chomen-7-yl) oxy)-*N*-((3-methyl-5-phenoxy-1phenyl-1*H*-pyrazole-4-yl)methylene)acetohydrazide (**7a**)

**White solid; Yield: 75%; m.p. 195–196 °C, **^**1**^**H NMR (400 MHz, DMSO-d**_**6**_**) *δ* ppm:** 11.38 (s, 1H, –NH), 7.80 (s, 1H, Ar–H), 7.66–7.71 (d, 1H, *J* = 8 Hz, Ar–H), 7.59–7.61 (d, 2H, *J* = 8 Hz, Ar–H), 7.43–7.47 (s, 2H, Ar–H), 7.32–7.36 (d, 3H, *J* = 8 Hz, Ar–H), 7.09–7.05 (t, 1H, *J* = 8 Hz, Ar–H), 6.99–7.01 (d, 2H, *J* = 8 Hz, Ar–H), 6.87–6.88 (d, 1H, *J* = 4 Hz, Ar–H), 6.77 (s, 1H, Ar–H), 6.22 (s, 1H, Ar–H), 4.88 (s, 2H, –OCH_2_), 2.50 (s, 3H, –CH_3_), 2.47 (s, 3H, –CH_3_). ^13^C NMR (100 MHz, DMSO-d_6_) *δ* ppm: 167.7 (C=O acetohydrazide linker), 164.2 (C=O coumarin lactone), 162.8 (C=O hydrazide), 161.7 (CH=N), 160.5, 158.9, 157.7, 156.6, 152.9, 150.4, 148.6 (Ar–C, Py), 147.3, 145.1, 140.3, 135.2, 133.6 (Ar–C, cou), 131.0, 127.2, 126.3, 124.6, 123.8 (Ar–CH, Py), 120.2, 117.8, 115.3, 107.2 (Ar–CH, cou), 62.4 (–OCH_2_–), 20.9 (CH_3_ *p*-tolyl), 18.7 (CH_3_ py). FTIR (ATR, $\tilde{\upnu }$, cm^−1^): 3298 (–NH), 3062, 3041 (C–H Ar), 2974, 2869 (C–H Ali), 1728 (C=O lactone), 1674 (–CONH), 1636 (CH=N), 1574, 1495 (C=C), 1418, 1376 (C–H), 1268, 1228, 1148 (C–O), 1029 (C–N), 968 (C–H), 932, 872 (Py ring), 786, 748 (C–H out-of-plane, substituted phenyl). ESI–MS (m/z): 508.53 [M + H]^+^ (base peak 509.39), Anal. calcd. for: C_29_H_24_N_4_O_5_; C, 68.49; H, 4.76; N, 11.02; O, 15.73%; Found: C, 68.46; H, 4.79; N, 11.05; O, 15.70%.

#### (*E*)-2-((4-methyl-2-oxo-2*H*-chomen-7-yl)oxy)-*N*-((3-methyl-5-(naphthalen-2-yloxy)-1-phenyl-1*H*-pyrazole-4-yl)methylene)acetohydrazide (**7b**)

**White solid; Yield: 79%; m.p. 197–198 °C, **^**1**^**H NMR (400 MHz, DMSO-d**_**6**_**) *δ* ppm:** 11.34 (s, 1H, –NH), 7.80 (s, 1H, Ar–H), 7.66–7.68 (d, 2H, *J* = 8 Hz, Ar–H,), 7.59–7.61 (d, 3H, *J* = 8 Hz, Ar–H), 7.43–7.47 (t, 2H, *J* = 8 Hz, Ar–H), 7.32–7.34 (d, 3H, *J* = 8 Hz, Ar–H), 7.05–7.09 (t, 1H, *J* = 8 Hz, Ar–H), 6.99–7.01 (d, 2H, *J* = 8 Hz, Ar–H), 6.86–6.88 (d, 1H, *J* = 4 Hz, Ar–H), 6.77 (s, 1H, Ar–H), 6.22 (s, 1H, Ar–H), 4.88 (s, 2H, –OCH_2_), 2.50 (s, 3H, –CH_3_), 2.40 (s, 3H, –CH_3_). ^13^C NMR (100 MHz, DMSO-d_6_) *δ* ppm: 168.9 (C=O acetohydrazide linker), 167.2 (C=O coumarin lactone), 164.9 (C=O hydrazide), 163.8 (CH=N), 160.4, 157.1, 156.9, 155.6, 154.9, 150.4 (Ar–C, Py), 148.6, 147.3, 145.1, 143.5 (Ar–C, coumarin), 141.3, 139.8, 136.1, 131.5 (Ar–CH, Py), 128.9, 127.1, 126.2, 122.3, 120.2 (Ar–CH, coumarin), 107.2, 65.4 (–OCH_2_), 21.3, 20.9 (–CH_3_ *p*-tolyl), 18.7 (–CH_3_ py). FTIR (ATR, $\tilde{\upnu }$, cm^−1^): 3295 (–NH), 3078, 3028 (C–H Ar), 2976, 2868 (C–H Ali), 1726 (C=O lactone), 1676 (–CONH), 1640 (CH=N), 1615, 1568 (C=C), 1465, 1425, 1385 (C–H), 1365, 1345 (C–N), 1320, 1280, 1255, (C–O), 1195 (C–N), 975 (C–H), 945, 915, 885, 865 (Py ring), 845, 785, 765, (C–H out-of-plane, naphthalene/phenyl substitution). ESI–MS (m/z): 558.59 [M + H]^+^ (base peak 559.38), Anal. cal. for: C_33_H_26_N_4_O_5_ C, 70.02; H, 4.69; N, 10.53; O, 14.95%; Found: C, 70.03; H, 4.70; N, 10.55; O 14.91%.

#### (*E*)-*N*-((3-methyl-1-phenyl-5-(*p*-tolyloxy)-1*H*-pyrazole-4-yl)methylene)-2-((4-methyl-2-oxo-2*H*-chomen-7-yl)oxy)-acetohydrazide (**7c**)

**White solid; Yield: 82%; m.p. 196–197 °C, **^**1**^**H NMR (400 MHz, DMSO-d**_**6**_**) *δ* ppm:** 11.32 (s, 1H, -NH), 7.79 (s, 1H, Ar–H), 7.67–7.69 (d, 1H, *J* = 8, Ar–H), 7.58–7.60 (d, 2H, *J* = 8 Hz, Ar–H), 7.43–7.47 (t, 3H, *J* = 8 Hz, Ar–H), 7.31–7.34 (t, 1H, *J* = 8 Hz, Ar–H), 7.11–7.13 (d, 2H, *J* = 8 Hz, Ar–H), 6.86–6.89 (d, 2H, *J* = 4 Hz, Ar–H), 6.72 (s, 1H, Ar–H), 6.22 (s, 1H, Ar–H), 4.86 (s, 2H, –OCH_2_), 2.50 (s, 3H, –CH_3_), 2.47 (s, 3H, CH_3_), 2.46 (s, 3H, CH_3_). ^13^C NMR (100 MHz, DMSO-d_6_) *δ* ppm: 168.9 (C=O acetohydrazide linker), 166.2 (C=O coumarin lactone), 163.9 (C=O hydrazide), 162.8 (CH=N), 160.4, 158.1, 154.6, 153.9 (Ar–C, Py), 148.6, 147.3, 144.1, 143.5, 141.3, 135.2 (Ar–C, coumarin), 131.0, 129.8, 128.1, 127.2 (Ar–CH, Py), 126.3, 125.6, 122.2, 117.8, 115.3 (Ar–CH, coumarin), 61.4 (–OCH_2_–), 20.9 (CH_3_ *p*-tolyl), 18.7 (CH_3_ py). FTIR (ATR, $\tilde{\upnu }$, cm^−1^): 3167 (–NH), 3050, (C–H Ar), 2360, 1887 (C–H Ali), 1712 (C=O lactone), 1673 (–CONH), 1603 (CH=N), 1600, 1587, 1561, 1496 (C=C), 1534 (N–H), 1495, 1411, 1391 (C–H), 1355 (C–N), 1316, 1297, 1192 (C–O), 947, 865, 828, 832 (Py ring), 809, 767, 689 (C–H out-of-plane, para-disubstituted phenyl). ESI–MS (m/z): 522.56 [M + H]^+^ (base peak 523.39), Anal. cal. for: C_30_H_26_N_4_O_5_ C, 68.95; H, 5.02; N, 10.72; O, 15.31%; Found: C, 68.90; H, 5.03; N, 10.77; O, 15.30%.

#### (*E*)-*N*-((3-methyl-1-phenyl-5-(m-tolyloxy)-1*H*-pyrazole-4-yl)methylene)-2-((4-methyl-2-oxo-2*H*-chomen-7-yl)oxy)-acetohydrazide (**7d**)

**White solid; Yield: 85%; m.p. 198–199 °C, **^**1**^**H NMR (400 MHz, DMSO-d**_**6**_**) *δ* ppm:** 11.38 (s, 1H, -NH), 7.79 (s, 1H, Ar–H), 7.66–7.68 (t, 1H, *J* = 8 Hz, Ar–H), 7.59–7.61 (d, 2H, *J* = 8 Hz, Ar–H), 7.43–7.47 (t, 2H, *J* = 8, Ar–H), 7.31–7.34 (t, 1H, *J* = 8 Hz, Ar–H), 7.19–7.23 (t, 1H, *J* = 8 Hz, Ar–H), 6.86–6.89 (m, 3H, Ar–H), 6.74–6.75 (s, 2H, Ar–H), 6.21 (s, 1H, Ar–H), 4.87 (s, 2H, –OCH_2_), 2.50 (s, 3H, –CH_3_), 2.48 (s, 3H, –CH_3_), 2.46 (s, 3H, –CH_3_). ^13^C NMR (100 MHz, DMSO-d_6_) *δ* ppm: 168.7 (C=O acetohydrazide linker), 161.2 (C=O coumarin lactone), 160.9 (C=O hyrdazide), 160.8 (CH=N), 159.7, 158.4, 154.1, 153.4, 148.6, 146.3 (Ar–C, Py), 145.1, 144.5, 143.3, 138.8, 137.1 (Ar–C, coumarin), 136.2, 135.0, 130.2, 129.1, 128.2 (Ar–CH, Py), 126.3, 122.6, 120.2, 116.8, 115.3, 113.8 (Ar–CH, coumarin), 65.4 (–OCH_2_–), 20.9 (–CH_3_ *p*-tolyl), 18.7 (–CH_3_ py)**. **FTIR (ATR, $\tilde{\upnu }$, cm^−1^): 3663 (–NH), 3156, 2986 (C–H Ar), 2360, 2078 (C–H Ali), 1707 (C=O lactone), 1677 (–CONH), 1637 (CH=N), 1602, 1592, 1502 (C=C), 1557 (N–H), 1482, 1411, 1392 (C–H), 1336 (C–N), 1298, 1204, 1155 (C–O), 928, 883, 831, 799, 766, 710, 692 (C–H out-of-plane, meta-disubstituted phenyl). ESI–MS (m/z): 522.56 [M + H]^+^ (base peak 523.38), Anal. cal. for : C_30_H_26_N_4_O_5_ C, 68.95; H, 5.02; N, 10.32; O, 15.85%; Found: C, 68.97; H, 5.04; N, 1O.33; O, 15.80%.

#### (*E*)-2-((4-methyl-2-oxo-2*H*-chomen-7-yl)oxy)-*N*-((3-methyl-5-(4-nitrophenoxy)-1-phenyl-1*H*-pyrazole-4-yl)methylene)acetohydrazide (**7e**)

**White solid; Yield: 76%; m.p. 198–199 °C, **^**1**^**H NMR (400 MHz, DMSO-d**_**6**_) ***δ* ppm:** 11.51 (s, 1H, -NH), 8.18–8.20 (d, 2H, *J* = 8 Hz, Ar–H), 7.83 (s, 1H, Ar–H), 7.57–7.64 (m, 3H, Ar–H), 7.44–7.48 (t, 2H, *J* = 8 Hz, Ar–H), 7.35–7.37 (t, 1H, *J* = 8 Hz, Ar–H), 7.27–7.29 (d, 2H, *J* = 8 Hz, Ar–H), 6.76–6.78 (d, 1H, *J* = 8 Hz, Ar–H), 6.62 (s, 1H, Ar–H), 6.22 (s, 1H, Ar–H), 4.70 (s, 2H, –OCH_2_), 2.50 (s, 3H, –CH_3_), 2.46 (s, 3H, –CH_3_). ^13^C NMR (100 MHz, DMSO-d_6_) *δ* ppm: 168.7 (C=O acetohydrazide linker), 165.2 (C=O coumarin lactone), 164.9 (C=O hydrazide), 163.8 (CH=N), 160.4, 158.1, 156.9, 154.6, 153.9 (Ar–C, Py), 150.4, 148.6, 146.3, 143.5, 140.3, 137.1, 135.6 (Ar–C, coumarin), 131.0, 130.2, 129.1, 127.2, 126.3 (Ar–CH, Py), 122.3, 120.2, 116.3, 113.8, 104.2 (Ar–CH, coumarin), 64.4 (–OCH_2_), 21.9 (–CH_3_ *p*-tolyl), 18.7 (–CH_3_ py). FTIR (ATR, $\tilde{\upnu }$~, cm^−1^): 3046 (–NH), 3069, 3072, (C–H Ar), 2360, 2325 (C–H Ali), 1714 (C=O lactone), 1677 (–CONH), 1643 (CH=N), 1604, 1597, 1504 (C=C), 1522 (N–H), 1522, 1349 (NO_2_), 1500, 1411, 1391 (C–H), 1347 (C–N), 1296, 1263, 1204, 1133 (C–O), 946, 917, 858, 835, 807, 795, 768, 691 (C–H out-of-plane, para-disubstituted phenyl). ESI–MS (m/z): 553.53 [M + H]^+^ (base peak 554.39), Anal. cal. for: C_29_H_23_N_5_O_7_ C, 67.90; H, 3.19; N, 10.60; O, 19.23%; Found: C, 67.93; H, 3.16; N, 10.62; O, 19. 21%.

#### (*E*)-2-((4-methyl-2-oxo-2*H*-cromen-7-yl)oxy)-*N*′-((2-phenoxyquinolin-3-yl)methylene) acetohydrazide (**11a**)

**White solid; Yield: 82%; m.p. 195–196 °C, **^**1**^**H NMR (400 MHz, DMSO-d**_**6**_**) *δ* ppm:** 10.30 (s, 1H, -NH), 8.87–8.95 (s, 2H, Ar–H), 8.51 (s, 1H, Ar–H), 8.02–8.12 (dd, 1H, *J* = 8 Hz, Ar–H), 7.65–7.75 (m, 3H, Ar–H), 7.58–7.60 (m, 1H, Ar–H), 7.47–7.53 (m, 3H, Ar–H), 7.29–7.31 (d, 2H, *J* = 8 Hz, Ar–H), 6.99–7.04 (m, 1H, Ar–H), 6.23–6.24 (s, 1H, Ar–H), 4.58 (s, 2H, –OCH_2_), 2.40 (s, 3H, –CH_3_). ^13^C NMR (100 MHz, DMSO-d_6_) *δ* ppm: 169.1 (C=O acetohydrazide linker), 166.6 (C=O coumarin lactone), 161.5 (C=O hydrazide), 153.8 (CH=N), 146.1, 142.6, 138.5, 136.2, 130.3 (Ar–C, Py), 139.2, 138.9, 137.9, 136.6, 136.9, 135.9, (Ar–C, coumarin) 127.3, 126.6, 125.8, 124.7, 122.4 (Ar–CH, Py), 121.9, 118.3, 115.7, 111.5, 102.5, 102.0 (Ar–CH, coumarin), 66.7 (–OCH_2_–,) 18.6 (–CH_3_ coumarin). FTIR (ATR, $\tilde{\upnu }$, cm^−1^): 3269 (–NH), 3167, 3057, (C–H Ar), 2360, 2376 (C–H Ali), 1723 (C=O lactone), 1680 (–CONH), 1641 (CH=N), 1625, 1616, 1578, 1521, 1504 (C=C), 1536 (N–H), 1487, 1474 (C–H), 1368, 1344 (C–N), 1326, 1298, 1150 (C–O), 965, 956, 878, 850, 808, 795, 778, 765, 745, 698 (C–H out-of-plane, quinoline/phenoxy substitution)*.* ESI–MS (m/z): 479.49 [M + H]^+^ (base peak 480.39), Anal. cal. for: C_28_H_21_N_3_O_5_ C, 70.14; H, 4.41; N, 8.76; O, 16.69%; Found: C, 70.15; H, 4. 40; N, 8.75; O, 16.70%.

#### (*E*)-2-((4-methyl-2-oxo-2*H*-cromen-7-yl)oxy)-*N*′-((2-(p-tolyloxy)quinolin-3-yl)methylene) acetohydrazide (**11b**)

**White solid; Yield: 78%; m.p. 197–198 °C, **^**1**^**H NMR (400 MHz, DMSO-d**_**6**_) ***δ* ppm:** 10.30 (s, 1H, -NH), 8.93 (s, 1H, Ar–H), 8.85–8.87 (d, 1H, *J* = 8 Hz, Ar–H), 8.51 (s, 1H, Ar–H), 8.01–8.10 (m, 1H, Ar–H), 7.64–7.75 (m, 1H, Ar–H), 7.57–7.59 (d, 1H, *J* = 8 Hz, Ar–H), 7.50–7.52 (s, 1H, Ar–H), 7.27–7.29 (d, 2H, J = 8 Hz, Ar–H), 7.17–7.18 (d, 2H, *J* = 8 Hz, Ar–H), 7.00–7.08 (m, 2H, Ar–H), 6.24 (s, 1H, Ar–H), 5.42 (s, 2H, –OCH_2_), 2.50 (s, 3H, –CH_3_), 2.40 (s, 3H, –CH_3_). ^13^C NMR (100 MHz, DMSO-d_6_) *δ* ppm: 169.1 (C=O acetohydrazide linker), 166.6 (C=O coumarin lactone) 161.6 (C=O hydrazude), 153.8 (CH=N) 146.7, 142.3, 138.5, 137.8, 136.9 (Ar–C, Py), 135.2, 134.9, 133.6, 132.5, 130.9, 1129.9 (Ar–C, coumarin), 129.8, 128.6, 127.8, 126.7, 125.4 (Ar–CH, Py), 124.9, 120.4, 119.5, 118.6, 117.5, 102.9 (Ar–CH, coumarin) 66.8 (–OCH_2_), 18.6 (–CH_3_ coumarin). FTIR (ATR, $\tilde{\upnu }$, cm^−1^): 3157 (–NH), 3047, 3035, (C–H Ar), 2260, 2329 (C–H Ali), 1707 (C=O lactone), 1670 (–CONH), 1641 (CH=N), 1622, 1602, 1575, 1503 (C=C), 1558 (N–H), 1482, 1464 (C–H), 1359, 1346 (C–N), 1313, 1234, 1203 (C–O), 975, 928, 882, 863, 830, 800, 767, 755, 757, 722, 686 (C–H out-of-plane, quinoline substitution). ESI–MS (m/z): 493.52 [M + H]^+^ (base peak 494.35), Anal. cal. for: C_29_H_23_N_3_O_5_ C, 70.58; H, 4.70; N, 8.51; O, 16.21%; Found: C, 70.55; H, 4.73; N, 8.52; O, 16.20%.

#### (*E*)-2-((4-methyl-2-oxo-2*H*-cromen-7-yl)oxy)-*N*′-((2-(m-tolyloxy)quinolin-3-yl)methylene) acetohydrazide (**11c**)

**White solid; Yield: 80%; m.p. 196–197 °C, **^**1**^**H NMR (400 MHz, DMSO-d**_**6**_) ***δ* ppm:** 10.80 (s, 1H, -NH), 9.94 (s, 1H, Ar–H), 8.95 (s, 1H, Ar–H), 8.49 (s, 1H, Ar–H), 8.09–8.12 (s, 1H, Ar–H), 8.02–8.04 (s, 1H, *J* = 8 Hz, Ar–H), 7.65–7.74 (m, 1H, Ar–H), 7.59–7.61 (d, 1H, *J* = 8 Hz, Ar–H), 7.48–7.53 (m, 2H, Ar–H), 7.34–7.38 (d, 1H, *J* = 8 Hz, Ar–H), 7.07–7.11 (m, 1H, Ar–H), 7.02–7.05 (t, 2H, *J* = 8 Hz, Ar–H), 6.22–6.23 (s, 1H, Ar–H), 5.42 (s, 2H, –OCH_2_), 2.50 (s, 3H, –CH_3_), 2.40 (s, 3H, –CH_3_). ^13^C NMR (100 MHz, DMSO-d_6_) *δ* ppm: 161.8 (C=O acetohydrazide linker), 160.4 (C=O coumarin lactone) 159.9 (C=O), 153.8 (CH=N), 146.7, 142.3, 139.5, 138.8, 136.9, 131.2 (Ar–C, Py) 129.6, 128.9, 127.6, 127.3, 126.2 (Ar–c, coumarin), 125.8, 122.6, 121.8, 120.7, 114.4, 113.9 (Ar–CH, Py), 112.4, 111.5, 111.6, 108.5, 102.9 (Ar–CH, coumarin), 65.8 (–OCH_2_–), 18.6 (CH_3_ coumarin). FTIR (ATR, $\tilde{\upnu }$, cm^−1^): 3291 (–NH), 2985, 2979, (C–H Ar), 2360, 2339 (C–H Ali), 1723 (C=O lactone), 1691 (–CONH), 1641 (CH=N), 1616, 1601, 1517, 1505 (C=C), 1534 (N–H), 1495, 1484 (C–H), 1368, 1342 (C–N), 1323, 1199, 1151 (C–O) 954, 919, 880, 862, 854, 791, 775, 746, 736, 722, 693, 671 (C–H out-of-plane, quinoline substitution). ESI–MS (m/z): 493.52 [M + H]^+^ (base peak 494.38), Anal. cal. for: C_29_H_23_N_3_O_5_ C, 70.58; H, 4.70; N, 8.51; O, 16.21%; Found: C 70.59; H, 4.71; N, 8.50; O, 16.20%.

#### (*E*)-2-((4-methyl-2-oxo-2*H*-cromen-7-yl)oxy)-*N*′-((2-(o-tolyloxy)quinolin-3-yl)methylene) acetohydrazide (**11d**)

**White solid; Yield: 87%; m.p**. **198–199 °C**, ^**1**^**H NMR (400 MHz, DMSO-d**_**6**_) ***δ* ppm:** 11.93 (s, 1H, -NH), 8.63 (s, 1H, Ar–H), 8.87–8.89 (d, 1H, *J* = 8 Hz, Ar–H), 8.56 (s, 1H, Ar–H), 8.02–8.11 (d, 1H, *J* = 8 Hz, Ar–H), 7.63–7.75 (m, 2H, Ar–H), 7.47–7.57 (m, 2H, Ar–H), 7.29–7.38 (m, 2H, Ar–H), 7.20–7.24 (m, 2H, Ar–H), 7.03–7.09 (m, 1H, Ar–H), 6.23–6.24 (s, 1H, Ar–H), 5.43 (s, 2H, –OCH_2_), 2.50 (s, 3H, –CH_3_), 2.41 (s, 3H, –CH_3_). ^13^C NMR (100 MHz, DMSO-d_6_) *δ* ppm: 169.1 (C=O acetohydrazide linker), 161.4 (C=O coumarin lactone), 155.0 (C=O hydrazide), 151.9 (CH=N), 146.7, 142.3, 138.5, 131.8, 129.9, 128.6 (Ar–C, Py), 127.9, 127.6, 127.5, 126.3, 126.4 (Ar–C, coumarin), 123.8, 121.6, 120.8, 119.7, 118.4, 116.9 (Ar–CH, Py), 115.4, 114.5, 112.8, 112.0, 102.9 (Ar–CH, coumarin), 67.8 (–OCH_2_–), 18.6 (–CH_3_ coumarin). FTIR (ATR, $\tilde{\upnu }$, cm^−1^): 2984 (–NH), 2360, 2358 (C–H Ar), 2346, 2340 (C–H Ali), 1726 (C=O lactone), 1678 (–CONH), 1644 (CH=N), 1625, 1612, 1522, 1517 (C=C), 1548 (N–H), 1489, 1440 (C–H), 1364, 1345 (C–N), 1327, 1193, 1175, 1148 (C–O), 915, 886, 849, 827, 795, 780, 759, 726, 700, 691, 681 (C–H out-of-plane, quinoline substitution). ESI–MS (m/z): 493.52 [M + H]^+^ (base peak 494.41), Anal. cal. for: C_29_H_23_N_3_O_5_ C, 70.58; H, 4.70; N, 8.51; O, 16.21%; Found: C, 70.60; H, 4.72; N, 8.49; O, 16.19%.

#### (*E*)-2-((4-methyl-2-oxo-2H-cromen-7-yl)oxy)-*N*′-((2-(4-nitrophenoxy)quinolin-3-yl)methylene)acetohydrazide (**11e**)

**White solid; Yield: 79%; m.p. 199–200 °C, **^**1**^**H NMR (400 MHz, DMSO-d**_**6**_**) *δ* ppm:** 11.95 (s, 1H, -NH), 9.02 (s, 1H, Ar–H), 8.94 (s, 2H, Ar–H), 8.83 (s, 1H, Ar–H), 8.47 (s, 1H, Ar–H), 8.35–8.37 (d, 2H, *J* = 8 Hz, Ar–H), 8.15–8.17 (d, 2H, *J* = 8 Hz, Ar–H), 8.07–8.09 (d, 1H, *J* = 8 Hz, Ar–H), 7.65–7.74 (m, 1H, Ar–H), 7.56–7.61 (m, 1H, Ar–H), 7.01–7.08 (m, 1H, Ar–H), 6.22–6.24 (s, 1H, Ar–H), 4.86 (s, 2H, –OCH_2_), 2.50 (s, 3H, –CH_3_). ^13^C NMR (100 MHz, DMSO-d_6_) *δ* ppm: 169.1 (C=O acetohydrazide linker), 161.8 (C=O coumarin lactone), 158.3 (C=O hydrazide), 153.8 (CH=N), 145.7, 144.3, 138.5, 137.8, 131.9 (Ar–C, Py), 129.2, 127.4, 127.3, 127.0, 126.6, 126.4 (Ar–C, coumarin), 126.0, 122.8, 119.7, 118.4, 116.9 (Ar–CH, Py), 113.4, 112.5, 111.6, 102.5, 108.9 (Ar–CH, coumarin), 65.8 (–OCH_2_–), 18.6 (–CH_3_ coumarin). FTIR (ATR, $\tilde{\upnu }$, cm^−1^): 2984 (–NH), 2360, 2351 (C–H Ar), 2340, 2332 (C–H Ali), 1726 (C=O lactone), 1678 (–CONH), 1643 (CH=N), 1612, 1604, 1585, 1521, 1507 (C=C), 1536 (N–H), 1520, 1347 (NO_2_), 1368, 1353 (C–N), 1345, 1286, 1193, 1148 (C–O), 1129, 1081, 1065 (C─H). 915, 892, 866, 849, 827, 802, 780, 759, 726, 701 (C–H out-of-plane, quinoline substitution). ESI–MS (m/z): 524.49 [M + H]^+^ (base peak 525.39), Anal. cal. for: C_28_H_20_N_4_O_7_ C, 64.12; H, 3.84; N, 10.68; O, 21.36%; Found: C, 64.10; H, 3.86; N, 10.70; O, 21.34%.

### Molecular docking studies

Molecular docking studies were performed using the Glide module of Schrödinger Suite (Schrödinger, LLC, New York, NY) to evaluate the binding interactions of the test compound with three target proteins: 4WMZ, 5FNR, and 4LOL. The three-dimensional crystal structures of the proteins were retrieved from the Protein Data Bank (PDB) at the RCSB. The protein structures were prepared using the Protein Preparation Wizard in Maestro, which included the addition of hydrogen atoms, assignment of bond orders, creation of disulfide bonds, and optimization of the hydrogen bonding network. Missing side chains and loops were filled using Prime. The protonation states of ionizable residues were assigned at pH 7.4 using PROPKA. Water molecules beyond 5 Å from heteroatoms were removed, and the structures were energy-minimized using the OPLS4 force field until the root mean square deviation (RMSD) of heavy atoms converged to 0.30 Å.

The ligand structure was prepared using the LigPrep module, where ionization states were generated at pH 7.4 ± 2.0 using Epik. Tautomers and stereoisomers were generated, and the ligand geometry was optimized using the OPLS4 force field. Receptor grid generation was performed using the Receptor Grid Generation panel in Glide. For protein 4WMZ, the grid box was centered on the active site residues with dimensions of 20 Å × 20 Å × 20 Å (X, Y, Z). For protein 5FNR, the grid box was defined with dimensions of 20 Å × 20 Å × 20 Å, centered at the catalytic site. Similarly, for protein 4LOL, a grid box of 20 Å × 20 Å × 20 Å was generated around the binding pocket. The scaling factor for van der Waals radii was set to 1.0 for receptor atoms with partial atomic charges less than 0.25.

Molecular docking was performed using Glide XP (Extra Precision) to obtain accurate binding pose predictions and reliable scoring. The ligand was docked flexibly, while the protein remained rigid. The best-docked poses were selected based on the Glide docking score (GScore), Glide energy (Emodel), and visual inspection of protein–ligand interactions. Post-docking analysis, including hydrogen bonding, hydrophobic interactions, and binding free energy calculations, was performed using the Glide XP Visualizer and the MM-GBSA method implemented in Prime^27,28^.

## Conclusion

This study successfully synthesized ten novel coumarin-pyrazole and coumarin-quinoline hybrids with optimized yield (88%) using multistep synthesis protocols. Compound **7a** emerged as the lead candidate, exhibiting superior antimicrobial activity (MIC: 25 μg/mL) and potent antioxidant potential (IC_50_: 20.23 mg/mL), outperforming standard drugs. Molecular docking revealed an exceptional binding affinity of **7b** for 4WMZ (− 9.71186 kcal/mol), driven by strategic hydrogen bonding and hydrophobic interactions. DFT calculations validated molecular stability with favorable reactivity descriptors (HOMO–LUMO gap: 3.8640 eV), while Mulliken charge, RDG, ELF, LOL, and thermodynamic analyses confirmed structural integrity. In silico ADMET profiling indicated favourable physicochemical and drug-like properties for most derivatives. However, uniformly high predicted genotoxicity scores (> 0.99) and moderate hERG liability were noted, necessitating comprehensive experimental safety evaluation before further advancement. This integrated approach establishes these coumarin hybrids as promising multifunctional therapeutic scaffolds, warranting further structure–activity optimisation and in vivo validation for clinical advancement.

## Supplementary Information

Below is the link to the electronic supplementary material.

Supplementary Material 1

## Data Availability

The datasets generated and analysed during the current study are available from the corresponding author on reasonable request. This includes optimized molecular structures, DFT calculation outputs, ADMET prediction data, and molecular docking results.
