# Supplementary material for: In silico DFT, ADMET and molecular docking studies of novel coumarin-linked pyrazole and quinoline derivatives as antimicrobial and antioxidant agents
Source: Sci Rep. 2026 May 27;16:24162. doi: 10.1038/s41598-026-52260-w (PMC13444106; doi:10.1038/s41598-026-52260-w)
Supplement: Supplementary file 1 [file 41598_2026_52260_MOESM1_ESM.docx]

**In Silico DFT, ADMET and Molecular Docking Studies of Novel Coumarin-Linked Pyrazole and Quinoline Derivatives as Antimicrobial and Antioxidant Agents**

Nargis H. Shaikh^a^, Jay B. Maheta^a^, Darshna K. Lakhnotra^a^, Yogesh O. Bhola^a^*, Prince A. Dave^a^, Ila M. Ram^a^, Dharmesh N. Sherathia^b^, Dharmesh K. Katariya^c^, Hardik B. Bhatt^c^, Swanti A. Jain^a^

^a^Department of Chemistry, Shri M.P. Pandya Science College, Lunawada, Mahisagar-389230, Gujarat, India.

^b^Department of Microbiology and Clinical technology, Dr. Subhas university, Junagadh- 362001, Gujarat, India.

^c^Department of Chemistry, Saurashtra University, Rajkot-360005, Gujarat, India.

^d^SET Science College & PG Centre, Bhakta Kavi Narsinh Mehta University, Junagadh-362001, Gujarat, India.

Corresponding author: dryobhola@gmail.com

**Supporting Information**

**Fig. S1:** ^1^H NMR spectra of final compound 7a

**Fig. S2:** ^1^H NMR spectra of final compound 7b

**Fig. S3:** ^1^H NMR spectra of final compound 7c

**Fig. S4:** ^1^H NMR spectra of final compound 7d

**Fig. S5:** ^1^H NMR spectra of final compound 7e

**Fig. S6:** ^1^H NMR spectra of final compound 11a

**Fig. S7:** ^1^H NMR spectra of final compound 11b

**Fig. S8:** ^1^H NMR spectra of final compound 11c

**Fig. S9:** ^1^H NMR spectra of final compound 11d

**Fig. S10:** ^1^H NMR spectra of final compound 11e

**Fig. S11:** ^13^C NMR spectra of final compound 7a

**Fig. S12:** ^13^C NMR spectra of final compound 7b

**Fig. S13:** ^13^C NMR spectra of final compound 7c

**Fig. S14:** ^13^C NMR spectra of final compound 7d

**Fig. S15:** ^13^C NMR spectra of final compound 7e

**Fig. S16:** ^13^C NMR spectra of final compound 11a

**Fig. S17:** ^13^C NMR spectra of final compound 11b

**Fig. S18:** ^13^C NMR spectra of final compound 11c

**Fig. S19:** ^13^C NMR spectra of final compound 11d

**Fig. S20:** ^13^C NMR spectra of final compound 11e

**Fig. S21:** Mass spectra of final compound 7a

**Fig. S22:** Mass spectra of final compound 7b

**Fig. S23:** Mass spectra of final compound 7c

**Fig. S24:** Mass spectra of final compound 7d

**Fig. S25:** Mass spectra of final compound 7e

**Fig. S26:** Mass spectra of final compound 11a

**Fig. S27:** Mass spectra of final compound 11b

**Fig. S28:** Mass spectra of final compound 11c

**Fig. S29:** Mass spectra of final compound 11d

**Fig. S30:** Mass spectra of final compound 11e

**Fig. S31:** IR spectra of final compound 7a

**Fig. S32:** IR spectra of final compound 7b

**Fig. S33:** IR spectra of final compound 7c

**Fig. S34:** IR spectra of final compound 7d

**Fig. S35:** IR spectra of final compound 7e

**Fig. S36:** IR spectra of final compound 11a

**Fig. S37:** IR spectra of final compound 11b

**Fig. S38:** IR spectra of final compound 11c

**Fig. S39:** IR spectra of final compound 11d

**Fig. S40:** IR spectra of final compound 11e

**Table S1:** Antimicrobial activity against various strains of synthesized compounds.

**Fig. S41:** Antimicrobial susceptibility testing results across five bacterial species showing differential growth patterns.

**Table S2:** Antioxidant activity against free radicals of synthesized compounds.

Concentration-dependent scavenging activity of Compounds 7a-e, 11a-e, and the standard drug ascorbic acid.

**Table S3:** Molecular docking of synthesized pyrazolone- Coumarin ring base 7(a-e) to 11(a-e) and amoxycillin drug with Stimulator of interferon genes protein (4LOL).

**Fig.S43:** 2D Molecular docking diagram (4LOL).

**Table S4:** Molecular docking of synthesized pyrazolone- Coumarin ring base 7(a-e) to 11(a-e) and Pefloxacin drug with lanosterol 14-alpha demethylase (4WMZ)

**Fig. S44:** 2D Molecular docking diagram (4WMZ)

**Table S5:** Molecular docking of synthesized pyrazolone- Coumarin ring base 7(a-e) to 11(a-e) and Ascorbic acid drug with malate dehydrogenase (5NFR)

**Fig.S45:** 2D Molecular docking diagram (5NFR)

**Table S6.** Mulliken Atomic Charges of Selected Atoms in the Optimized Structure

**Fig. S1:** ^1^H NMR spectra of final compound 7a

**Fig. S2:** ^1^H NMR spectra of final compound 7b

**Fig. S3:** ^1^H NMR spectra of final compound 7c

**Fig. S4:** ^1^H NMR spectra of final compound 7d

**Fig. S5:** ^1^H NMR spectra of final compound 7e

**Fig. S6:** ^1^H NMR spectra of final compound 11a

**Fig. S7:** ^1^H NMR spectra of final compound 11b

**Fig. S8:** ^1^H NMR spectra of final compound 11c

**Fig. S9:** ^1^H NMR spectra of final compound 11d

**Fig. S10:** ^1^H NMR spectra of final compound 11e

**Fig. S11:** ^13^C NMR spectra of final compound 7a

**Fig. S12:** ^13^C NMR spectra of final compound 7b

**Fig. S13:** ^13^C NMR spectra of final compound 7c

**Fig. S14:** ^13^C NMR spectra of final compound 7d

**Fig. S15:** ^13^C NMR spectra of final compound 7e

**Fig. S16:** ^13^C NMR spectra of final compound 11a

**Fig. S17:** ^13^C NMR spectra of final compound 11b

**Fig. S18:** ^13^C NMR spectra of final compound 11c

**Fig. S19:** ^13^C NMR spectra of final compound 11d

**Fig. S20:** ^13^C NMR spectra of final compound 11e

 **Fig. S21:** Mass spectra of final compound 7a

**Fig. S22:** Mass spectra of final compound 7b

**Fig. S23:** Mass spectra of final compound 7c

**Fig. S24:** Mass spectra of final compound 7d

**Fig. S25:** Mass spectra of final compound 7e

**Fig. S26:** Mass spectra of final compound 11a

**Fig. S27:** Mass spectra of final compound 11b

**Fig. S28:** Mass spectra of final compound 11c

**Fig.S29:** Mass spectra of final compound 11d

**Fig. S30:** Mass spectra of final compound 11e

**Fig. S31:** IR spectra of final compound 7a

**Fig. S32:** IR spectra of final compound 7b

**Fig. S33:** IR spectra of final compound 7c

 **Fig. S34:** IR spectra of final compound 7d

**Fig. S35:** IR spectra of final compound 7e

 **Fig. S36:** IR spectra of final compound 11a

**Fig. S37:** IR spectra of final compound 11b

 **Fig. S38:** IR spectra of final compound 11c

 **Fig. S39:** IR spectra of final compound 11d

**Fig. S40:** IR spectra of final compound 11e

**Table S1:** Antimicrobial activity against various strains of synthesized compounds.

| **Sample ID/Name** | **B. subtilis (ATCC 6633)** | **S. aureus (ATCC 6538)** | **E. coli (433)** | **A. baumannii (1425)** | **A. niger** |
| --- | --- | --- | --- | --- | --- |
| 7a | 25 | 25 | 25 | 50 | 25 |
| 7b | 50 | 25 | 50 | 50 | 50 |
| 7c | 200 | 50 | 100 | 50 | 50 |
| 7d | 50 | 100 | 100 | 100 | 50 |
| 7e | 50 | 100 | 100 | 100 | 100 |
| 11a | 200 | 50 | 100 | 50 | 50 |
| 11b | 100 | 50 | 100 | 50 | 50 |
| 11c | 100 | 50 | 100 | 100 | 50 |
| 11d | 100 | 50 | 100 | 100 | 100 |
| 11e | 100 | 50 | 50 | 100 | 50 |
| Amoxycillin (Std.) | 12.5 | 25 | 200 | 25 | — |
| Fluconazole (Std.) | — | — | — | — | 100 |

**Fig. S41:** Antimicrobial susceptibility testing results across five bacterial species showing differential growth patterns.

**Table S2:** Antioxidant activity against free radicals of synthesized compounds.

| Compounds | Scavenging activity (IC_50_ mg mL^-1^) |
| --- | --- |
| 7a | 20.23 ± 0.29 |
| 7b | 24.20 ± 0.64 |
| 7c | 27.16 ± 0.72 |
| 7d | 32.44 ± 0.29 |
| 7e | 45.40 ± 0.87 |
| 11a | 28.60 ± 0.35 |
| 11b | 37.65 ± 0.21 |
| 11c | 39.92 ± 0.23 |
| 11d | 34.85 ± 0.47 |
| 11e | 47.43 ± 0.50 |
| Ascorbic acid | 25.80 ± 0.94 |

**Concentration-dependent scavenging activity of Compounds 7a-e, 11a-e, and the standard drug ascorbic acid.**

**Table S3:** Molecular docking of synthesized pyrazolone- Coumarin ring base 7(a-e) to 11(a-e) and amoxycillin drug with Stimulator of interferon genes protein (4LOL).

| **Compound code** | **Docking score (kcal/mol)** | **Interacting amino acids** | | | |
| --- | --- | --- | --- | --- | --- |
|  |  | **Hydrophobic interactions** | **Polar interactions** | **H-bond** | **Others** |
| 7a | -4.61564 | LEU 180, PHE 383, PRO 384. PRO 278, ALA 279, MET 281, ILE 287 | ASN 283, HIE 163 | ARG 284, (H-bond with coumarin lactone) | ARG 175, ARG 387, (charged positive), ASP 288, GLU 176, (charged negative) GLY 177, GLY 385, ARG 282 (Pi- cation with pyrazole and coumarin) |
| 7b | -4.2113 | LEU 180, PHE 383, PRO 384. PRO 278, VAL 289, MET 281, ILE 287 | ASN 283, HIE 163 | ARG 387, (H-bond with coumarin CO) | ARG 175, ARG 284, (charged positive), ASP 288, GLU 176, (charged negative) GLY 177, GLY 385, ARG 282 (Pi- cation with pyrazole and coumarin) |
| 7c | -3.76746 | LEU 180, PHE 383, PRO 384. PRO 278, ALA 279, MET 281, ILE 287 | ASN 283, HIE 163 | ARG 284, (H-bond with coumarin lactone) | ARG 175, ARG 387, (charged positive), ASP 288, GLU 176, GLU 386,(charged negative) GLY 177, GLY 385, ARG 282 (Pi- cation with pyrazole) |
| 7d | -3.92476 | LEU 180, PHE 383, PRO 384. PRO 278, VAL 289, MET 281, ILE 287 | ASN 283, HIE 163 | ARG 387, (H-bond with coumarin CO) | ARG 175, ARG 284, (charged positive), ASP 288, GLU 176, GLU 386, (charged negative) GLY 177, GLY 385, ARG 282 (Pi- cation with pyrazole and coumarin) |
| 7e | -3.73837 | LEU 180, PHE 383, PRO 384. PRO 278, VAL 289, MET 281, ILE 287 | ASN 283, HIE 163 | ARG 284, (H-bond with coumarin lactone), ARG 387, (H-bond with coumarin CO) | ARG 175, (charged positive), ASP 288, GLU 176, (charged negative) GLY 177, GLY 385, ARG 282 (Pi- cation with pyrazole and coumarin) |
| 11a | -2.63702 | LEU 180, PHE 383, PRO 384. PRO 278, ALA 279, MET 281, ILE 287 | ASN 283 | GLY 177 (H-bond with coumarin lactone) | ARG 175, ARG 282, LYS 245 (charged positive), ASP 288, GLU 176(charged negative), GLY 177, GLY 385, ARG 284 (Pi- cation with quinoline) |
| 11b | -3.79063 | LEU 180, PHE 383, PRO 384. PRO 278, ALA 279, MET 281, ILE 287 | ASN 283, HIE 281 | ARG 282, (H-bond with coumarin oxygen) | ARG 175 (charged positive), GLU 176 (charged negative), GLY 177, GLY 385 |
| 11c | -3.88281 | LEU 180, PHE 383, PRO 384. PRO 278, ALA 279, MET 281, ILE 287 | ASN 283, HIE 281 | ARG 282, (H-bond with coumarin oxygen) | ARG 175 (charged positive), GLU 176 (charged negative), GLY 177, GLY 385 |
| 11d | -3.7571 | LEU 180, PHE 383, PRO 384. PRO 278, ALA 279, MET 281, ILE 287 | ASN 283, HIE 281 | ARG 284, (H-bond with coumarin lactone) | ARG 175, ARG 387, (charged positive), GLU 176, (charged negative) GLY 177, GLY 385, ARG 282 (Pi- cation with coumarin) |
| 11e | -4.14943 | LEU 180, PHE 383, PRO 384. PRO 278, VAL 288, MET 281, ILE 287 | ASN 283, HIE 281 | ARG 387, (H-bond with coumarin CO) | ARG 282,  (charged positive), GLU 386, GLU 176, (charged negative) GLY 177, GLY 385, ARG 175 (salt bridge with NO_2_ group of quinoline) |
| Amoxycillin | -4.00365 | PRO 278, MET 281, ILE 287, PRO 384, PHE 383, LEU 180 | ASN 283 | ARG 175, HIE 163 (H- bond with NH_3_ and OH, respectively | ARG 387, ARG 282 (charged positive), GLU 176 (charged negative), GLY 177, GLY 385 |

**Fig.S43:** 2D Molecular docking diagram (4LOL).

**Table S4:** Molecular docking of synthesized pyrazolone- Coumarin ring base 7(a-e) to 11(a-e) and Pefloxacin drug with lanosterol 14-alpha demethylase (4WMZ)

| **Compound**  **code** | **Docking score (kcal/mol)** | **Interacting amino acids** | | | |
| --- | --- | --- | --- | --- | --- |
|  |  | **Hydrophobic interactions** | **Polar interactions** | **H-bond** | **Others** |
| 7a | -9.52145 | VAL 242, PHE 241, LEU 95, LEU 96, PRO 238, PHE 236, MET 100, TYR 140, TYR 126, LEU 129, LEU 380, LEU 383, MET 509, PHE 384, PHE 506, TYR 72, VAL 70, ALA 69 | THR 507, SER 508, SER 382, HIE 381, HIS 468 | - | ARG 98, ARG 385 (charged positive), GLY 73, GLY 314 |
| 7b | -9.71186 | PHE 463, ILE 471, PHE 475, LEU 312, VAL 311, LEU 283, LEU 158, LEU 212, TYR 140, ILE 139, VAL 154, LEU 147, MET 509, PHE 134, PHE 236, LEU 383, LEU 380, TYR 126, LEU 129 | THR 318, HIS 468, THR 130 | CYS 470 (H-bond with Schiff base group) | ARG 469, LYS 151( charged positive), GLY 315, GLY 314, GLY 472, GLY 310 |
| 7c | -9.30025 | VAL 311, LEU 212, LEU 158, VAL 154, LEU 307, PHE 475, LEU 147, LEU 380, CYS 470, ILE 471, LEU 383, ILE 139, TYR 126, MET 509, PHE 236, PHE 134, PHE 113, LEU 129 | THR 130, HIS 468, GLN 150, THR 318 | - | LYS 151 (charged positive), GLY 315, GLY 314, GLY 472, TYR 140 (Pi-Pi stacking with coumarin) |
| 7d | -9.75359 | PHE 506, TYR 72, VAL 70, ALA 69, VAL 242, ILE 239, PRO 238, LEU 95, LEU 96, PHE 236, MET 100, TYR 126, LEU 129, ILE 139, TYR 140, PHE 134, LEU 380, LEU 383, MET 509, PHE 384 | THR 507, SER 508, SER 382, HIE 381, HIS 468, THR 13O | - | ARG 98 (charged positive), GLY 73, PHE 241 (Pi-Pi stacking with pyrazole) |
| 7e | -8.66217 | MET 509, TYR 126, LEU 129, PHE 236, PHE 134, ILE 139, VAL 311, ALA 476, PRO 462, PHE 463, LEU 374, PHE 113, LEU 383, LEU 380, CYS 470, PRO 379, ILE 471 | THR 130, HIE 378, HIS 468, THR 318 | - | ARG 469 (charged positive), GLY 310, GLY 314, GLY 315, GLY 464, GLY 465, GLY 472, ARG 385 (salt bridge with NO_2_ group of pyrazole), TYR 140 (Pi-Pi stacking with coumarin) |
| 11a | -9.29941 | PHE 384, LEU 383, PHE 236, PRO 138, LEU 95, LEU 380, LEU 96, PHE 241, VAL 242, MET 100, TYR 72, MET 509, PHE 506, LEU 129, PHE 134, TYR 140 | THR 130, THR 506, SER 382, HIE 381, | TYR 126 (H-bond with coumarin oxygen) | ARG 98 (charged positive), GLY 314, GLY 73 |
| 11b | -9.69644 | TYR 72, ALA 69, LEU 95, LEU 96, VAL242, PHE 241, LEU 380, PRO 238, LEU 383, PHE 236, PHE 134, ILE 139, TYR 140, TYR 126, CYS 470, PHE 113, PHE 463, MET 509, PHE 506 | HIE 381, HIS 468 | - | ARG 385, ARG 469 (charged positive), GLY 73, GLY 314, GLY 310, GLY 465, GLY 464, SER 382 (salt bridge with Schiff base ), PHE 384 (Pi-Pi stacking with coumarin) |
| 11c | -9.76427 | MET 509, LEU 307, PHE 236, PHE 134, VAL 311, LEU 147, TYR 140, ILE 139, VAL 154, LEU 158, LEU 212, PHE 475, ILE 471, CYS 470, LEU 380, LEU383, LEU 129 | THR 130, HIS 468, GLN 150, THR 318 | - | LYS 151 ( charged positive), GLY 310, GLY 314, GLY 315, GLY 472, TYR 126 (Pi-Pi stacking with coumarin) |
| 11d | -9.07891 | LEU 158, LEU 212, PHE 475, VAL 154, LEU 307, LEU 147, VAL 311, LEU 380, LEU 139, TYR 140, LEU 383, LEU 129, MET 509, PHE 236, PHE 134, CYS 470, ILE 471 | HIS 468, THR 318, THR 130 | - | LYS 151 (charged positive), GLY 310, GLY 314, GLY 315, GLY 472, TYR 126 (Pi-Pi stacking with coumarin) |
| 11e | -8.60497 | PHE 236, MET 509, LEU 129, ILE 139, VAL 311, PHE 134, LEU 383, PHE 113, PHE 463, LEU 380, PRO 379, PRO 462, LEU 374, ALA 476, PHE 475, ILE 471, CYS 470, VAL 154, LEU 158, LEU 212, | THR 130, HIS 468, HIE 378, THR 318, THR 322 | - | ARG 385 (charged positive), GLY 310, GLY 315, GLY 314, GLY 464, GLY 472, TYR 140, TYR 126 (Pi-Pi stacking with coumarin) |
| Fluconazole | -6.69497 | PHE 236, PHE 134, VAL 311, TYR 140, ILE 139, LEU 307, LEU 147, ILE 471, CYS 470, VAL 154 | THR 130, GLN 150, HIS 468 | LYS 151(H- bond with triazole) | ARG 469 (charged positive), GLY 314, GLY 310, LYS 151(Pi-cation with aromatic ring), PHE 134 (Pi-Pi stacking with triazole) |

**Fig. S44:** 2D Molecular docking diagram (4WMZ)

**Table S5:** Molecular docking of synthesized pyrazolone- Coumarin ring base 7(a-e) to 11(a-e) and Ascorbic acid drug with malate dehydrogenase (5FNR)

| **Compound code** | **Docking score (kcal/mol)** | **Interacting amino acids** | | | |
| --- | --- | --- | --- | --- | --- |
|  |  | **Hydrophobic interactions** | **Polar interactions** | **H-bond** | **Others** |
| 7a | -8.95446 | VAL 418, ALA 556, ALA 510, VAL 463, VAL 465, ALA 466, VAL 512, CYS 513, VAL 467, VAL 514, VAL 561, VAL 608, ALA 607, VAL 369, CYS 368, ALA 366, LEU 365, VAL 604, VAL 467 | THR 560 | - | ARG 415 (charged positive) , GLY 462, GLY 509, GLY 464, GLY 511, GLY 558, GLY 367, GLY 605, GLY 364, GLY 603, GLY 419 |
| 7b | -8.84944 | VAL 463, VAL 465, VAL 467, ALA 510, CYS 513, VAL 514, LEU 514, ILE 559, VAL 561, VAL 369, CYS 368, VAL 420, VAL 608, VAL 607, VAL 604, VAL 418, ALA 366, LEU 365, ALA 556, LEU 557, ILE 559 | - | VAL 512 (H- bond with Schiff base group ), | ARG 415 (charged positive), GLY 464, GLY 462, GLY 511, GLY 509, GLY 605, GLY 419, GLY 367, GLY 603, GLY 417, GLY 364, GL 558, THR 560 (salt bridge with coumarin) |
| 7c | -8.62839 | VAL 608, VAL 420, CYS 368, ALA 607, VAL 369, VAL 604, VAL 418, ALA 366, LEU 365, VAL 561, ILE 559, LEU 557, ALA 556,VAL 463, ALA 510, VAL 465, ALA 466, VAL 467, VAL 512, CYS 513, VAL 514, LEU 515 | - | - | ARG 415 (charged positive), GLY 605, GLY 603, GLY 419, GLY 417, GLY 367, GLY 364, GLY 558, GLY 462, GLY 509, GLY 464, GLY 511, THR 560 (salt bridge with pyrazole) |
| 7d | -8.83413 | VAL 561, VAL 512, CYS 513, VAL 514, LEU 515,V ALA 510, VAL 608, ALA 607, VAL 606, VAL 604, ALA 556, LEU 365, VAL 463, ALA 366, CYS 368, VAL 369, VAL 467, ALA 466, VAL 465, VAL 418 | THR 560 | ILE 559, GLY 367 (H- bond with Schiff base and coumarin CO, respectively) | ARG 415 (charged positive), GLY 603, GLY 605, GLY 558, GLY 509, GLY 364, GLY 462, GLY 464, GLY 419 |
| 7e | -8.91649 | VAL 608, ALA 607, VAL 420, VAL 369, CYS 368, VAL 604, VAL 418, ALA 366, LEU 365, VAL 561, ILE 559, LEU 557, ALA 556, VAL 463, VAL 465, ALA 466, VAL 467, VAL 512, ALA 510, CYS 513, VAL 514 | - | - | ARG 415, (charged positive), GLY 605, GLY 603, GLY 471, GLY 419, GLY 367, GLY 364, GLY 558, GLY 462, GLY 464, GLY 509, GLY 511, THR 560 (salt bridge with pyrazole) |
| 11a | -7.81352 | CYS 513, VAL 514, VAL 608, VAL 369, CYS 368, ALA 607, VAL 604, ALA 366, LEU 365, VAL 467, ALA 466, VAL 465, VAL 463, VAL 561, ILE 559, VAL 418, ILE 416 | THR 560 | VAL 606, GLY 367 (H-bond with Schiff base group) | ARG 415 (charged positive), GLY 558, GLY 325, GLY 464, GLY 462, GLY 417, GLY 509, GLY 364, GLY 603, GLY 605 |
| 11b | -8.21527 | VAL 512, VAL 465, VAL 463, TYR 334, LEU 365, ALA 366, VAL 418, ILE 416, VAL 604, LEU 557, ALA 556, TYR 525, TYR 572, PHE 577 | ASN 382, SER 363, SER 602, SER 555, GLN 530 | VAL 606, GLY 367 (H- bond with coumarin lactone) | GLY 509, GLY 462, GLY 464, GLY 364, GLY 417, GLY 605, GLY 603, GLY 558, ARG 415 (Pi-cation with quinoline) |
| 11c | -8.85216 | VAL 604, VAL 465, ALA 366, LEU 365, VAL 512, VAL 463, LEU 557, ALA 556, ILE 416, TYR 525, PHE 577, TYR 334 | ASN 414, ASN 382, SER 363, SER 506 | VAL 606, GLY 367 (H- bond with coumarin lactone) | ARG 483 (charged positive), GLY 417, GLY 605, GLY 603, GLY 464, GLY 364, GLY 558, GLY 462, GLY 509, ARG 415 (Pi- cation with aromatic ring), PHE 478 ( Pi-Pi stacking with quinoline) |
| 11d | -8.03104 | VAL 512, VAL 604, TYR 525, VAL 465, ALA 366, VAL 463, LEU 365, LEU 557, VAL 418, ALA 556, ILE 416, TYR 334, TYR 572, PHE 577 | ASN 382, SER 555, SER 363, SER 602, GLN 530 | VAL 606, GLY 367 (H- bond with coumarin lactone), | GLY 509, GLY 603, GLY 605, GLY 464, GLY 462, GLY 364, GLY 558, GLY 417, ARG 415 ( Pi -cation with quinoline) |
| 11e | -8.09103 | TYR 334, ILE 416, VAL 418, PHE 478, TYR 525, VAL 463, VAL 465, VAL 512, VAL 606, VAL 604, ALA 366, LEU 365, LEU 557, ALA 556, ILE 461 | SER 508, ASN 382, SER 363 | VAL 606, GLY 367, ARG 483 (H -bond with coumarin lactone and quinoline NO_2_ group, respectively) | GLY 417, GLY 509, GLY 462, GLY 464, GLY 605, GLY 603, GLY 364, GLY 558, ARG 415 ( Pi -cation with quinoline) |
| Ascorbic acid | -5.05538 | CYS 368, ALA 366, ILE 416, VAL 463, VAL 465, CYS 513 ILE 559 | - | VAL 512, VAL 418, VAL 606, GLY 367 (H-bond with cyclic lactone and 3 OH) | GLY 605, GLY 417, GLY 464 |

**Fig.S45** 2D Molecular docking diagram (5FNR)

**Table S6.** Mulliken Atomic Charges of Selected Atoms in the Optimized Structure.

| **Sr. No** | **Atom** | **Charge (Q/e)** | **Sr. No** | **Atom** | **Charge (Q/e)** |
| --- | --- | --- | --- | --- | --- |
| 1 | C | -0.21013 | 32 | C | -0.19992 |
| 2 | C | 0.334516 | 33 | C | 0.308758 |
| 3 | C | -0.19065 | 34 | C | -0.20107 |
| 4 | C | 0.344139 | 35 | C | -0.18752 |
| 5 | C | -0.04594 | 36 | C | -0.18933 |
| 6 | C | -0.20155 | 37 | C | -0.1873 |
| 7 | O | -0.58916 | 38 | C | -0.19277 |
| 8 | C | 0.648655 | 39 | H | 0.201767 |
| 9 | C | -0.29849 | 40 | H | 0.227315 |
| 10 | C | 0.083308 | 41 | H | 0.201447 |
| 11 | C | -0.6047 | 42 | H | 0.215214 |
| 12 | O | -0.4718 | 43 | H | 0.220672 |
| 13 | O | -0.57816 | 44 | H | 0.220922 |
| 14 | C | -0.20145 | 45 | H | 0.215357 |
| 15 | C | 0.650764 | 46 | H | 0.240492 |
| 16 | N | -0.55012 | 47 | H | 0.24053 |
| 17 | O | -0.50304 | 48 | H | 0.367904 |
| 18 | N | -0.32938 | 49 | H | 0.250091 |
| 19 | C | 0.044998 | 50 | H | 0.216041 |
| 20 | C | -0.18188 | 51 | H | 0.223914 |
| 21 | C | 0.716792 | 52 | H | 0.219625 |
| 22 | N | -0.70259 | 53 | H | 0.225735 |
| 23 | N | -0.34454 | 54 | H | 0.190937 |
| 24 | C | 0.360653 | 55 | H | 0.187043 |
| 25 | C | -0.60552 | 56 | H | 0.191152 |
| 26 | C | 0.34822 | 57 | H | 0.22159 |
| 27 | O | -0.60585 | 58 | H | 0.2071 |
| 28 | C | -0.18325 | 59 | H | 0.195016 |
| 29 | C | -0.18855 | 60 | H | 0.189577 |
| 30 | C | -0.18444 | 61 | H | 0.194538 |
| 31 | C | -0.1893 | 62 | H | 0.213619 |
